# Collagen-VI supplementation by cell transplantation improves muscle regeneration in Ullrich congenital muscular dystrophy model mice

**DOI:** 10.1186/s13287-021-02514-3

**Published:** 2021-08-09

**Authors:** Nana Takenaka-Ninagawa, Jinsol Kim, Mingming Zhao, Masae Sato, Tatsuya Jonouchi, Megumi Goto, Clémence Kiho Bourgeois Yoshioka, Rukia Ikeda, Aya Harada, Takahiko Sato, Makoto Ikeya, Akiyoshi Uezumi, Masashi Nakatani, Satoru Noguchi, Hidetoshi Sakurai

**Affiliations:** 1 Department of Clinical Application, Center for iPS Cell Research and Application (CiRA), Kyoto University, 53 Kawahara-cho, Shogoin, Sakyo-ku, Kyoto, 606-8507 Japan; 2 Department of Anatomy, Fujita Health University, Toyoake, Aichi 470-1192 Japan; 3 Muscle Aging and Regenerative Medicine, Research Team for Geriatric Medicine, Tokyo Metropolitan Institute of Gerontology, Tokyo, 173-0015 Japan; 4 Division for Therapies against Intractable Diseases, Institute for Comprehensive Medical Science (ICMS), Fujita Health University, Toyoake, Aichi 470-1192 Japan; 5 Department of Neuromuscular Research, National Institute of Neuroscience, Department of Clinical Development, Translational Medical Center, National Center of Neurology and Psychiatry, Kodaira, Tokyo, 187-8551 Japan

**Keywords:** Induced pluripotent stem cells, Mesenchymal stromal cells, Skeletal muscle regeneration, Ullrich congenital muscular dystrophy, COL6 related disease

## Abstract

**Background:**

Mesenchymal stromal cells (MSCs) function as supportive cells on skeletal muscle homeostasis through several secretory factors including type 6 collagen (COL6). Several mutations of *COL6A1*, *2*, and *3* genes cause Ullrich congenital muscular dystrophy (UCMD). Skeletal muscle regeneration deficiency has been reported as a characteristic phenotype in muscle biopsy samples of human UCMD patients and UCMD model mice. However, little is known about the COL6-dependent mechanism for the occurrence and progression of the deficiency. The purpose of this study was to clarify the pathological mechanism of UCMD by supplementing COL6 through cell transplantation.

**Methods:**

To test whether COL6 supplementation has a therapeutic effect for UCMD, in vivo and in vitro experiments were conducted using four types of MSCs: (1) healthy donors derived-primary MSCs (pMSCs), (2) MSCs derived from healthy donor induced pluripotent stem cell (iMSCs), (3) COL6-knockout iMSCs (COL6KO-iMSCs), and (4) UCMD patient-derived iMSCs (UCMD-iMSCs).

**Results:**

All four MSC types could engraft for at least 12 weeks when transplanted into the tibialis anterior muscles of immunodeficient UCMD model (*Col6a1*KO) mice. COL6 protein was restored by the MSC transplantation if the MSCs were not COL6-deficient (types 1 and 2). Moreover, muscle regeneration and maturation in *Col6a1*KO mice were promoted with the transplantation of the COL6-producing MSCs only in the region supplemented with COL6. Skeletal muscle satellite cells derived from UCMD model mice (*Col6a1*KO-MuSCs) co-cultured with type 1 or 2 MSCs showed improved proliferation, differentiation, and maturation, whereas those co-cultured with type 3 or 4 MSCs did not.

**Conclusions:**

These findings indicate that COL6 supplementation improves muscle regeneration and maturation in UCMD model mice.

**Supplementary Information:**

The online version contains supplementary material available at 10.1186/s13287-021-02514-3.

## Background

Ullrich congenital muscular dystrophy (UCMD) is a slowly progressive disease characterized by muscle weakness from birth, muscle atrophy, proximal joint contracture, distal joint hyperextension, and ultimately respiratory failure (1, 2). At present, no effective cure has been found. Patient biopsies revealed a deficiency of type 6 collagen (COL6) in the muscles, and later it was found that *COL6A1*, *COL6A2*, and *COL6A3* mutations cause defective extracellular microfibril assembly (3–5).

COL6 is a fibril molecule with a molecular weight of about 500 kDa and is widely distributed in the extracellular matrix (ECM) of systemic tissues including skeletal muscle (6–9). It interacts with fibrous collagen such as collagen 1 and many kinds of ECM and basement membrane proteins (10–12). Although one of the major functions of COL6 protein is to connect the basement membrane to fibrous connective tissue, it has also been reported to interact with integrin, cytokines, and growth factors and is thought to be involved in cell proliferation, differentiation, and regeneration (13–24). Moreover, COL6 deficiency alters the ECM structure and biomechanical properties and leads to mitochondrial defects (25–30), decreased autophagy (31–33), and impaired muscle regeneration (34, 35).

Previous case reports on UCMD patients have described histological phenotypes (36–39). The pathological findings of UCMD are dystrophic changes that consist of connective tissue proliferation, degenerated necrotic fibers, marked variation in fiber size, and type 1 fiber atrophy and pre-dominance (36). In 2017, a UCMD model mouse (*Col6a1*^GT/GT^ mouse) completely deficient of COL6 was established (40). In the skeletal muscle of this model and another UCMD model, the number of medium- and large-diameter muscle fibers approximated that in wild-type (WT) mice, but the number of muscle fibers with extremely small diameters was much larger (40, 41), a phenotype consistent with the pathology of UCMD patients.

In previous studies, fibroblast (35) and human adipose tissue-derived MSC (ADSC) (34) transplantations were done to examine if COL6 supplementation can regulate the regeneration of toxin-injured skeletal muscle in UCMD model mice. Although short-term effects, such as the enhancement of satellite cell proliferation and regenerating myofibers, were demonstrated on intact and damaged muscles, long-term effects, such as the enhancement of myofiber maturation and enlargement of fiber size, are still unknown in fibroblast transplantations (35). In the case of ADSC transplantation, while long-term engraftment was demonstrated on injured muscle, the therapeutic effects for muscle regeneration and maturation were not (34). In addition to COL6 secretion, other ECM and soluble factors produced by MSCs in skeletal muscle play important roles in the differentiation, regeneration, and homeostasis of myogenic cells and the maintenance of skeletal muscle fibers (42–45). Additionally, MSCs show abnormal properties in UCMD mouse models (40). Therefore, it remains unclear whether the therapeutic effect of cell transplantation is due to COL6 supplementation or healthy MSC supplementation.

The aims of this study are (1) to clarify the long-term effects of COL6 supplementation by cell transplantation and (2) to clarify whether the therapeutic effects of MSC transplantation are due to COL6 supplementation or other factors. Therefore, in the present study, using induced pluripotent stem cell (iPSC) technology (46), the short-term and long-term effects of COL6 on the intact muscle of UCMD model mice in cell transplantation therapy were directly examined by comparing fully functional MSCs with MSCs deficient in COL6 secretion (COL6-deficient MSCs). As a result, the transplantation of functional MSCs promotes the regeneration and maturation of muscle fibers by supplementing COL6, but the transplantation of COL6-deficient MSCs does not. Similarly, co-culture experiments with healthy MSCs promoted the proliferation, fusion, and maturation of skeletal muscle satellite cells (MuSCs) derived from *Col6a1*KO mice, whereas co-culture experiments with COL6-deficient MSCs did not.

## Methods

### Generation of *Col6a1*KO mice

Heterozygous mice were produced by crossing NOD.Cg-Prkdc^scid^Il2rg^tm1Wjl^/SzJ (NSG mouse; severe immunodeficient) with *Col6a1*^GT/GT^ mice (40). Subsequently, by heterozygous multiplication, *Col6a1*^GT/GT^/NSG mice were identified by genotyping the resulting litter population and used in the experiments as *Col6a1*KO (immunodeficient UCMD model) mice. For the genotyping, genome DNA was extracted from the tail of each mouse. To select homozygous *Col6a1*^GT/GT^/*Il2r*^*-/-*^ mice, genomic PCR was done by genotyping Fw-Rv primer pairs for *Col6a1* and *Il2r* (the primers are listed in Table S4).

### Induction of iMSCs from iPSCs

iMSCs (induced mesenchymal stromal cells) were derived from iPSCs through neural crest cells (NCCs), as reported previously(47). Even though cells in the stroma of skeletal muscle tissue are called MSCs, fibro adipogenic progenitor cells and mesenchymal progenitor cells, the cells used in this study were defined as MSCs according to (47). Human iPSCs were cultured on an iMatrix-511 (Nippi, Tokyo, Japan)-coated cell culture plate or dish in StemFit AK03N (Ajinomoto, Tokyo, Japan) and then induced to differentiate into NCCs as described previously (47). Briefly, the induction and maintenance of NCCs were performed using previously reported CDMi medium (48), which contains Iscove's modified Dulbecco’s medium/Ham’s F-12 1∶1, 1x chemically defined lipid concentrate (GIBCO, Grand Island, NY, USA), 15 μg/mL apo-transferrin (Sigma-Aldrich, St. Louis, MO, USA), 450 μM monothioglycerol (Sigma-Aldrich), 5 mg/mL purified BSA (99% purified by crystallization; Sigma-Aldrich), 7 μg/mL Insulin (FUJIFILM Wako, Osaka, Japan), and penicillin/streptomycin (Invitrogen, Carlsbad, CA, USA). To induce NCCs, 10 μM SB431542 (SB) (584-77601, Sigma-Aldrich) and 1 μM CHIR99021 (CHIR) (FUJIFILM Wako) were added to the CDMi medium. The cells were trypsinized, and p75+ (CD271+) NCCs were then sorted by FACS AriaII (BD Biosciences, San Jose, CA, USA) according to the manufacturer’s protocol (the antibodies used are listed in Table S3). EGF (059-07873, FUJIFILM Wako) and basic FGF (47079000, Oriental Yeast CO., LTD, NIB, Tokyo, Japan) were added to CDMi medium to maintain NCCs.

The expanded NCCs (passage number, 3-10) were seeded onto fibronectin-coated plates at a density of 1 × 10^4^ cells/cm^2^ in CDMi medium supplemented with 10 μM SB, 20 ng/mL EGF, and 20 ng/mL basic FGF. The medium was replaced the next day with α-MEM (nacalai tesque, Kyoto, Japan) supplemented with 5 ng/mL basic FGF and 10% fetal bovine serum (FBS) (556-33865, FUJIFILM Wako). The morphology of the cells started to change approximately 4 days after the induction. Passages were performed every 3 days using Accutase (nacalai tesque) at a density 5 × 10^5^ cells in a 10-cm dish (Corning, Corning, NY, USA) coated with fibronectin and cultured with αMEM supplemented with 5 ng/mL basic FGF and 10% FBS and maintained at 37 °C in 5% CO_2_.

### Isolation of primary (p)MSCs

Non-dystrophic healthy muscle samples were obtained from the gluteus medius muscles of female subjects undergoing total hip arthroplasty. The methods for dissociating cells from the muscle samples and sorting pMSCs (PDGFRα+) are described elsewhere (49). Briefly, muscle samples were transferred to PBS and digested with 0.2% type II collagenase (Worthington, Columbus, OH, USA). Muscle slurries were filtered through a cell strainer (BD Biosciences). Cells were resuspended in growth medium (GM) consisting of DMEM (Sigma-Aldrich) supplemented with 20% FBS, 1% penicillin-streptomycin (Katayama Chemical, Osaka, Japan), and 2.5 ng/mL basic FGF, seeded onto a collagen I-coated dish (Iwaki, Shizuoka, Japan), and maintained at 37 °C in 5% CO_2_ and 3% O_2_. The cells were trypsinized, and PDGFRα+ pMSCs were sorted using FACS Vantage SE (BD Biosciences) or MoFlo Astrios (Beckman Coulter, Brea, CA, USA) (the antibodies and reagents used are listed in Table S3). The collected pMSCs were cultured in GM and maintained at 37 °C in 5% CO_2_ and 3% O_2_.

### MSC transplantation into *Col6a1*KO mice

*Col6a1*KO mice (4–6-weeks old) were anesthetized for surgery with 3% Forane inhalant liquid (AbbVie, North Chicago, IL, USA). Human iMSCs or pMSCs were suspended in αMEM (2 × 10^6^ cells/50 μL) and injected into the mice using a 27G micro-syringe (Myjector syringe; Terumo, Tokyo, Japan) at the center of the tibialis anterior (TA) muscles. TSG6 (R&D systems, Minneapolis, MN, USA) was co-injected at a concentration of 2 μg/L to improve the cell engraftment efficiency (50). The same amount of αMEM was injected into the TA muscle of the limb opposite the limb into which the cells were transplanted and used as a comparison target for histological analysis.

### Statistical analysis

Animals were excluded from the study only if their health status was compromised; for instance, if they had visible wounds due to fighting. The investigators were blinded to the outcome assessments. A one-way analysis of variance (ANOVA), Welch’s ANOVA, and the Kruskal-Wallis test were conducted to assess differences among three groups or more. Unpaired Student’s *t* tests were calculated to assess differences among two groups.

Differences were considered significant for p values ≤ 0.05. All statistical analyses were performed using EZR version 1.37 (Jichi Medical University Saitama Medical Center) (51).

### Other materials and methods

Other materials and methods are described in the Supplemental Methods.

## Results

### Comparison of healthy iMSCs, KO-iMSCs, and pMSCs

**Fig. 1:**
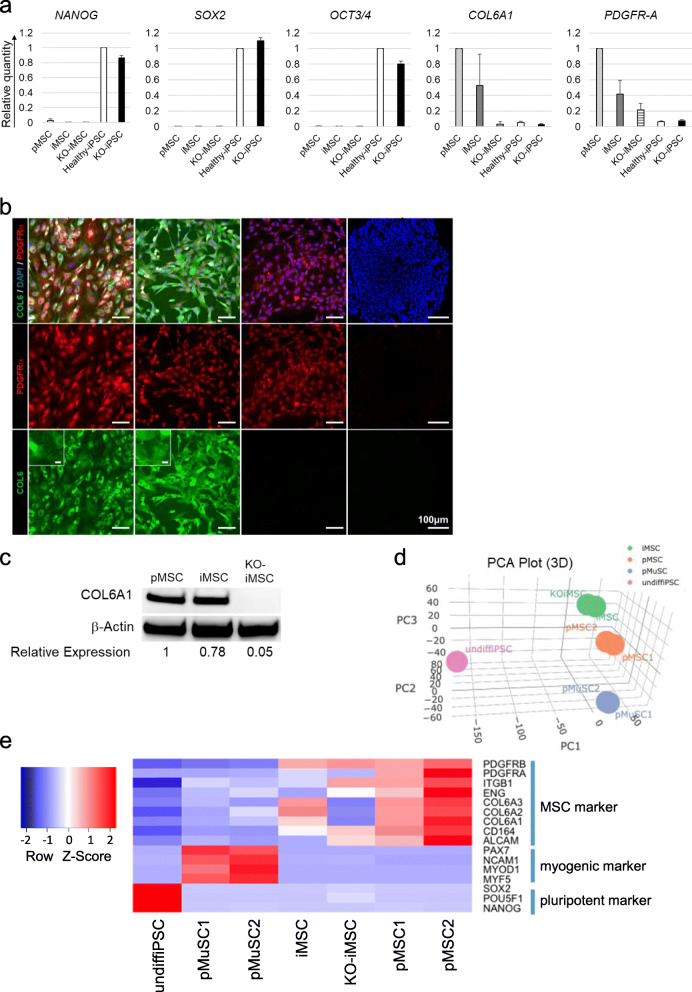
Characteristics of pMSCs, iMSCs, and KO-iMSCs. **a** The expression of marker genes. The mRNA expression level of each gene was analyzed by RT-qPCR in pMSCs derived from skeletal muscle, iMSCs, KO-iMSCs, undifferentiated healthy iPSCs, and *COL6A1*KO-iPSCs. Levels are shown relative to the level in healthy iPSCs for the pluripotency markers *NANOG*, *SOX2*, and *OCT3/4* or in pMSCs for the MSC markers *COL6A1* and *PDGFR-A*. Data are shown as the mean ± SD. *n* = 3. **b** Immunofluorescence images of pMSCs, iMSCs, KO-iMSCs, and healthy iPSCs. The inserts in the lower panels are higher magnifications and show the fibril structure. Scale bars, 100 μm, and 10 μm (inserts). **c** Western blots of COL6A1 protein expression in pMSCs, iMSCs, and KO-iMSCs. β-Actin was used as the control. Average fold changes between iMSCs, KO-iMSCs, and pMSCs are indicated below. **d** PCA plot showing the clustering of cell populations based on the transcriptomes. Proportion of variance: PC1 = 43.764%, PC2 = 19.746%, PC3 = 17.682%. **e** Heatmap of MSC markers, myogenic markers, and pluripotency markers

To elucidate whether the supplementation of COL6 is indispensable for the improvement of muscle regeneration and maturation in UCMD model mice, *COL6A1* knockout iPSCs were created using the CRISPR-Cas9 System (Figure S1) and differentiated into KO-iMSCs. Properties of the iMSCs, KO-iMSCs, and pMSCs were compared. Unlike iPSCs, iMSCs had a low expression of pluripotency marker genes (Figure 1a). The expression of *PDGFR-A*, an MSC marker gene, and its protein were highly expressed in all three MSC types (Figure 1a, b). *COL6A1* mRNA was highly expressed in pMSCs and iMSCs, but barely in iPSCs or KO-iMSCs (Figure 1a). COL6 was expressed intracellularly and further secreted after forming a fibril structure outside the cell in pMSCs and iMSCs, but not in KO-iMSCs or iPSCs (Figure 1b, bottom row). Western blotting analysis verified that COL6 protein was expressed in pMSCs and iMSCs, but not in KO-iMSCs (Figure 1c). Flow cytometer analysis confirmed pMSCs and both types of iMSCs uniformly expressed MSC-positive markers including CD105, CD73, CD44, and CD201 (49) but not the negative marker CD45 (Figure S2). A comprehensive gene expression analysis by RNA-seq showed that iMSCs and KO-iMSCs have properties closer to pMSCs than to undifferentiated iPSCs or MuSCs derived from healthy human skeletal muscle tissue (Figure 1d). In addition, iMSCs, KO-iMSCs, and pMSCs showed a high expression of MSC markers, but a low expression of pluripotency markers and myogenic markers (Figure 1e). These results confirmed that iMSCs are a comparable cell population with properties similar to skeletal muscle derived-MSCs, and KO-iMSCs have similar character except for COL6 expression.

### COL6 was supplemented by the local injection of iMSCs/pMSCs into *Col6a1*KO mouse muscle

**Fig. 2:**
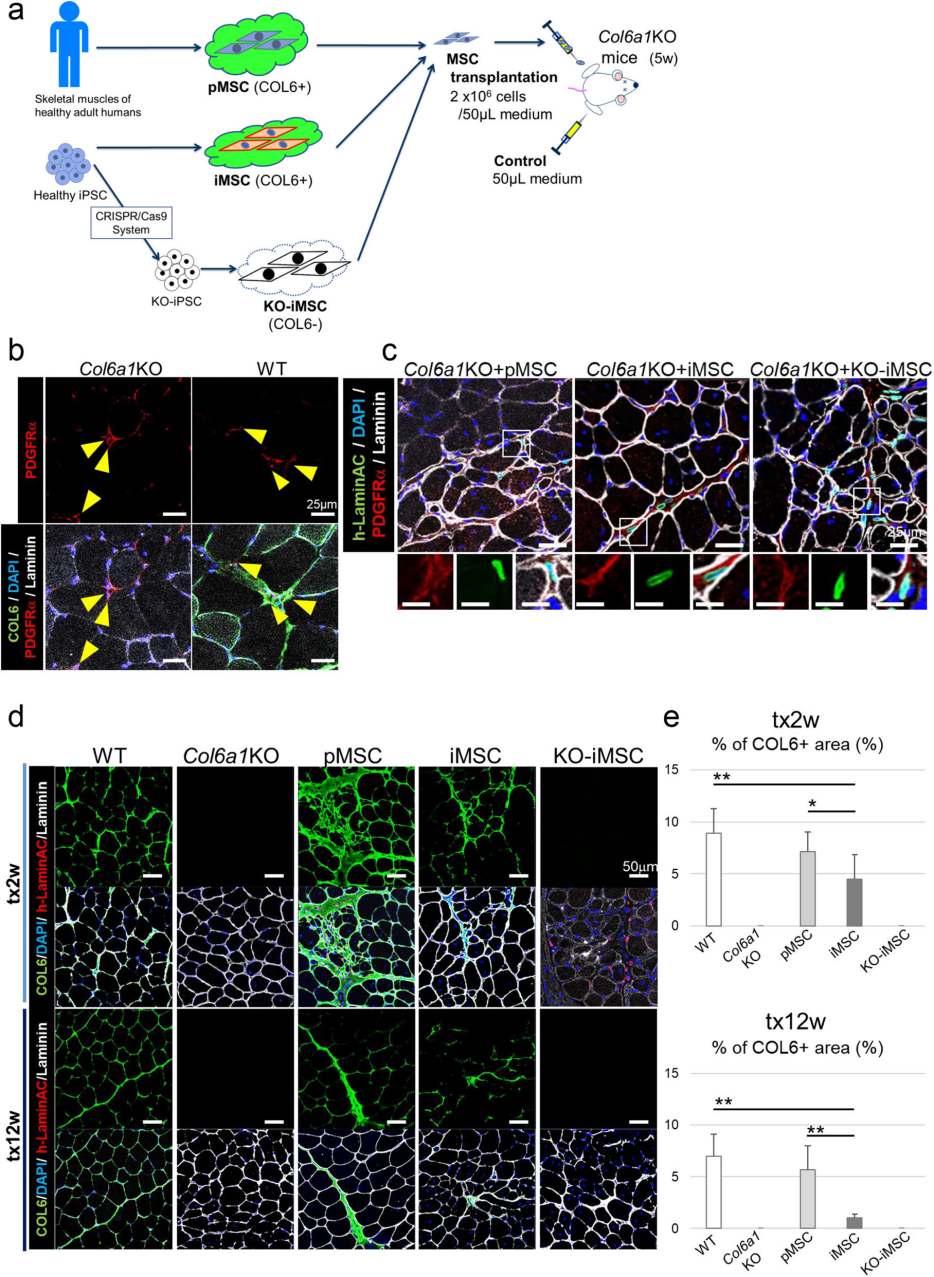
Cell engraftment and COL6 supplementation in TA muscles of *Col6a1*KO mice after cell transplantation. **a** Schematic representation of the cell transplantation into the TA muscle of *Col6a1*KO mice. **b** Sectional images of TA muscles in a *Col6a1*KO mouse and a WT mouse. Arrowheads indicate PDGFRα+ cells. **c** Sectional images of TA muscles 2 weeks after the cell transplantation. Boxed areas in the upper panels are shown with higher magnification in the lower panels. Scale bars, 25 μm (upper) and 12 μm (lower). **d** Sectional images of TA muscles at 2 weeks (upper) and 12 weeks (lower) after medium injection (WT and *Col61*KO) or cell transplantation. **e** Quantitative data of the COL6 positive area per whole TA CSA 2 weeks (tx2w) or 12 weeks (tx12w) after the transplantation. Data are shown as the mean ± SD. For tx2w, *n* = 17 (WT), *n* = 10 (medium; *Col6a1*KO), *n* = 9 (pMSCs), *n* = 12 (iMSCs), and *n* = 7 (KO-iMSCs); for tx12w, *n* = 17 (WT), *n* = 11 (medium; *Col6a1*KO), *n* = 6 (pMSCs, iMSCs, and KO-iMSCs each). *P < 0.05. **P < 0.01

pMSCs, iMSCs, and KO-iMSCs were transplanted into the TA muscles of *Col6a1*KO mice intramuscularly (Figure 2a). Two or 12 weeks after the transplantation, histological analysis of the TA muscles was performed. Without cell transplantation, PDGFRα-positive MSCs were found in the interstitium of skeletal muscle tissue, but COL6 protein was deficient (Figure 2b), consistent with a previous report (40). On the other hand, in the skeletal muscle tissue of WT mice of the same age, COL6 protein wrapped each muscle fiber (Figure 2b). Two weeks after the transplantation, human Lamin A/C-positive transplanted cells were engrafted in the interstitium, and PDGFRα expression was maintained in the TA muscle sections of *Col6a1*KO mice (Figure 2c). Furthermore, COL6 protein was detected around human Lamin A/C-positive transplanted cells in the TA muscle tissues transplanted with pMSCs and iMSCs at 2 and 12 weeks after the transplantation (Figure 2d). COL6 protein secreted from the transplanted cells wrapped around the host muscle fibers in a manner consistent with skeletal muscle tissue in WT mice. On the other hand, in the muscle tissues transplanted with KO-iMSCs, COL6 protein was not detected at all (Figure 2d). At 2 weeks after the transplantation, 5% of the area of TA muscles that received iMSC or pMSC transplantation expressed COL6 protein (Figure 2d, e). However, after 12 weeks, the COL6 expression was only maintained in the pMSC-transplanted limbs, whereas in the iMSC transplanted limb, the size of the COL6-positive area had decreased (Figure 2d, e). In addition, the transplanted cells in all cases did not remain at the transplanted site but migrated to disperse (Figure S3a). No pathological fibrosis was seen in all transplanted limbs (Figure S3b). The number of human Lamin A/C-positive transplanted pMSCs, iMSCs, and KO-iMSCs greatly decreased between 1 and 2 weeks after the transplantation (Figure S4a). A time-course histological analyses showed that the COL6 expression level was reduced at 4 weeks after the transplantation of pMSCs or iMSCs and maintained until 24 weeks (Figure S4b, c).

### COL6 supplementation by iMSCs/pMSCs transplantation improves the impairment of muscle maturation in *Col6a1*KO mice

**Fig. 3:**
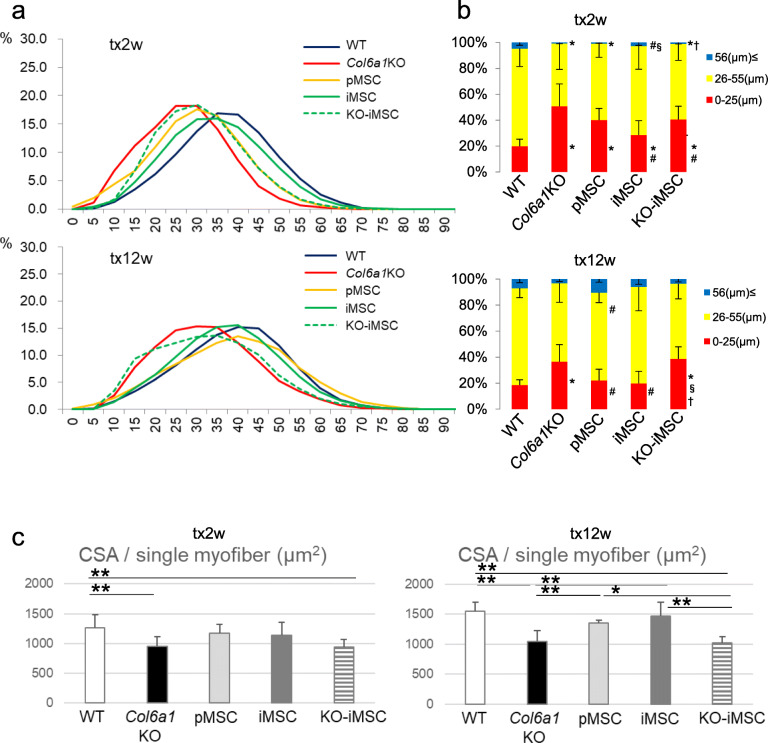
Histological changes in skeletal muscle tissue of *Col6a1*KO mice by COL6 supplementation. **a** Quantification of myofiber CSAs in the TA muscles of *Col6a1*KO mice 2 weeks (tx2w) and 12 weeks (tx12w) after medium injection or cell transplantation. Control WT mice are the same age as *Col6a1*KO mice. The *x*-axis shows the short axis of the muscle fiber CSA in μm. **b** The short axis of the myofiber CSAs in **a** shown as band graphs (blue, 56 μm ≤ CSA short axis; yellow, 26 μm ≤ CSA short axis ≤ 55 μm; red, CSA short axis ≤ 25 μm). *P < 0.05 vs WT, ^#^*P* < 0.05 vs *Col6a1*KO, ^§^*P* < 0.05 vs pMSC, ^†^*P* < 0.05 vs iMSC. **c** CSA per single myofiber in TA muscles. Data are shown as the mean ± SD. For tx2w, *n* = 15 (WT), *n* = 10 (medium; *Col6a1*KO), *n* = 9 (pMSCs), *n* = 12 (iMSCs), and *n* = 7 (KO-iMSCs); for tw12w, *n* = 17 (WT), *n* = 11 (medium; *Col6a1*KO), *n* = 6 (pMSCs, iMSCs, and KO-iMSCs each). *P < 0.05. **P < 0.01

It has been reported that the proportion of small-diameter skeletal muscle fibers is greater in *Col6a1*^GT/GT^ mice than in WT mice (40). This phenotype was true for the *Col6a1*KO mice used in this study (Figure 3a, b). However, at 2 weeks after the transplantation, the proportion of small-diameter fibers decreased in muscle tissue transplanted with iMSCs and pMSCs (Figure 3a, b). This trend continued at 12 weeks after the transplantation, and the proportion of large-diameter fibers increased. The average cross-sectional area (CSA) of single muscle fibers was increased in TA muscles transplanted with pMSCs and iMSCs at 12 weeks (Figure 3c). The number of TA muscle fibers tended to increase with iMSC or KO-iMSC transplantation at 2 weeks (Figure S5a), and at 12 weeks, the number of myofibers in pMSC- and iMSC-transplanted TA muscles reached that of WT mice (Figure S5a). The whole CSA and muscle wet weight of iMSC- and pMSC-transplanted TA muscles increased compared with *Col6a1*KO muscles, whereas KO-iMSC-transplanted TA muscles showed no difference with *Col6a1*KO muscle (Figure S5b, c). These results indicated that COL6 supplementation by COL6-producing pMSCs/iMSCs increased the muscle fiber diameter in *Col6a1*KO TA muscle.

### COL6 supplementation by iMSCs/pMSCs transplantation promotes muscle regeneration in *Col6a1*KO mice

**Fig. 4:**
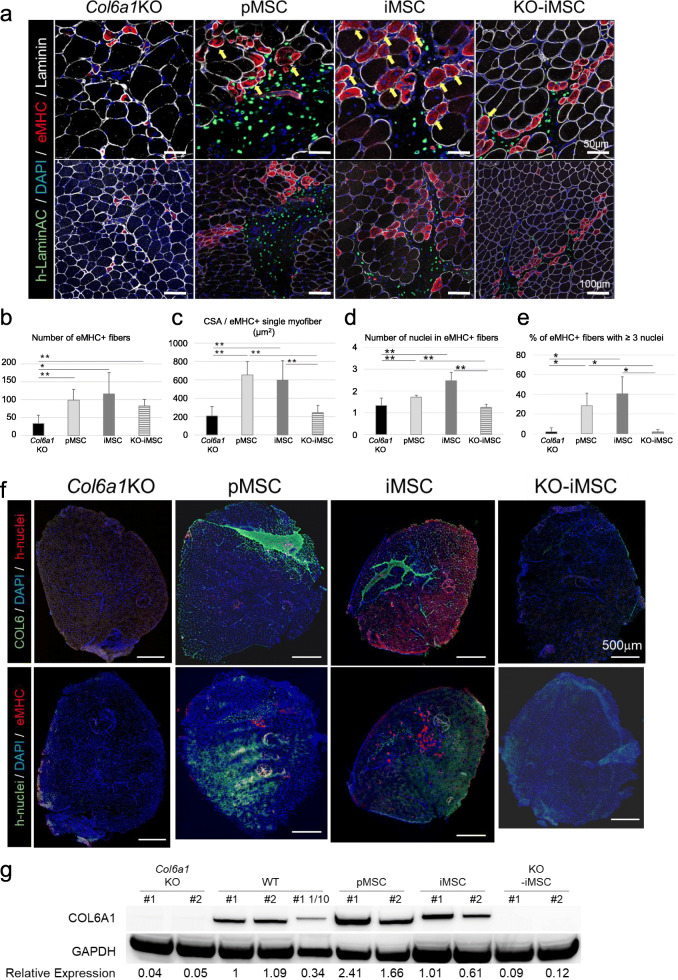
Small-diameter regenerating muscle fibers (eMHC+) in *Col6a1*KO mice were matured by COL6 supplementation. **a** Sectional images of TA muscles 1 week after medium injection (*Col6a1*KO) or cell transplantation. Yellow arrows indicate multi nuclei eMHC+ fibers. **b** Number of eMHC-positive fibers 1 week after the transplantation. **c** CSA per single eMHC+ TA muscle fiber 1 week after the transplantation. **d** Number of nuclei in eMHC+ TA muscle fibers 1 week after the transplantation. **e** % of eMHC+ TA muscle fibers with more than three nuclei 1 week after the transplantation. **f** Sectional images of whole TA muscle 1 week after medium injection (*Col6a1*KO) or cell transplantation. Two serial sections were stained. **g** Western blots for COL6A1 protein expression in pMSC-, iMSC-, and KO-iMSC-transplanted TA muscle tissue samples of *Col6a1*KO mice and TA muscle tissue samples of WT mice. GAPDH was used as the control. Data are the mean ± SD. *n* = 20 (medium; *Col6a1*KO), *n* = 10 (iMSCs), and 6 (pMSCs and KO-iMSCs each). *P < 0.05. **P < 0.01

In a previous study using muscle biopsies from UCMD patients, regenerating muscle fibers labeled with an antibody for embryonic myosin heavy chain (eMHC), which is transiently upregulated in regenerating fibers, were limited to only small diameters (36). Consistently, all eMHC-positive fibers were small in the muscles of *Col6a1*KO mice, suggesting that the fibers remained immature. In all transplanted TA muscles, eMHC-positive fibers gathered in the areas of the transplanted cells (Figure 4a). The number of eMHC-positive fibers significantly increased in MSC-transplanted TA muscles (Figure 4b). The CSA of eMHC-positive fibers was increased in COL6-producing pMSC/iMSC-transplanted muscles (Figure 4c). In iMSC-transplanted TA muscles, regenerating muscle fibers were seen to be multinucleated (Figure 4a, arrows). The number of nuclei per muscle fiber was increased in iMSC-transplanted TA muscle (Figure 4d), and more than 40% of regenerating muscles had more than three nuclei, but the percentage was only 5% in *Col6a1*KO mice (Figure 4e). An analysis using serial sections showed that eMHC-positive fibers were greatly matured in the region supplemented with COL6 protein (Figure 4f). A western blotting analysis showed pMSC- or iMSC-transplanted *Col6a1*KO muscles restored COL6 protein to the same or higher levels than in WT muscle (Figure 4g). These results indicate that COL6-producing pMSCs/iMSCs promote the regeneration of eMHC-positive small-diameter muscle fibers in *Col6a1*KO TA muscles.

The therapeutic effect of the transplantation in adult mice with advanced symptoms was also examined. Transplantation of iMSCs into the TA muscles of 30-week-old *Col6a1*KO mice showed that COL6 supplementation and muscle regeneration were correlated (Figure S6).

To analyze whether the supplementation of COL6 protein is enough to promote muscle regeneration, recombinant human COL6 (rh-COL6) protein was injected alone into *Col6a1*KO muscles (Figure S7a). The rh-COL6 protein was slightly detected in muscle tissue 1 week after the injection (Figure S7b), but almost all of it had disappeared at 2 weeks. In addition, eMHC-positive regenerating myofibers at 1 week after the administration did not increase and remained small (Figure S7c). As a result, the injection of rh-COL6 protein did not affect either the number of eMHC-positive fibers or CSA.

### COL6 supplementation by iMSCs/pMSCs transplantation promotes the activation of MuSCs and the proliferation of myoblasts

**Fig. 5:**
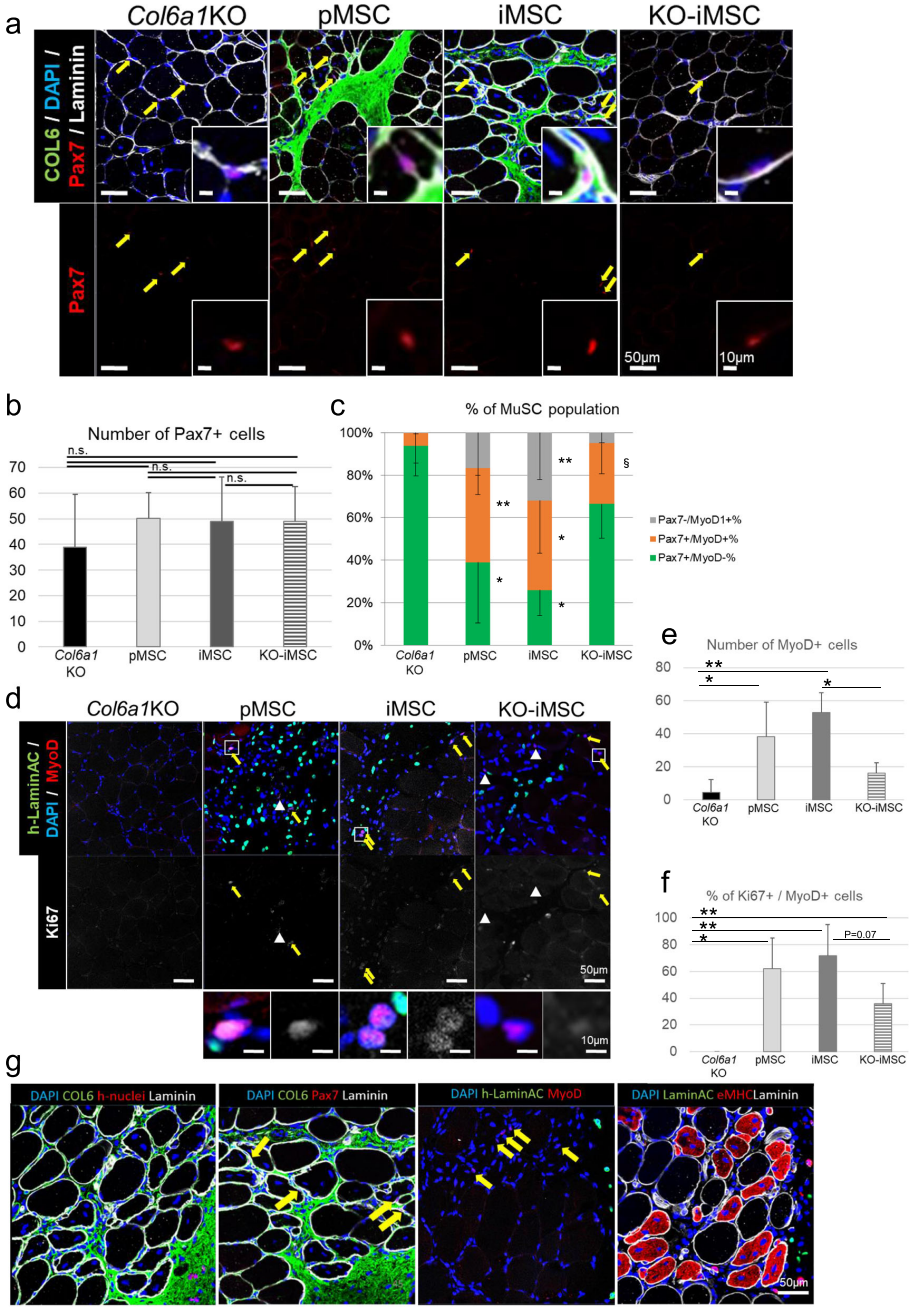
Verification of MuSC proliferation and differentiation and regenerative muscle maturation by COL6 supplementation. **a** Sectional images of TA muscle 1 week after medium injection (*Col6a1*KO) or cell transplantation. Yellow arrows indicate Pax7+ nuclei. The inserts are higher magnifications. **b** Number of Pax7+ cells 1 week after the transplantation. *n* = 20 (medium; *Col6a1*KO), *n* = 6 (pMSCs), *n* = 10 (iMSCs), and *n* = 6 (KO-iMSCs). **c** Quantification of Pax7 + MyoD–, Pax7 + MyoD+, and Pax7–MyoD+ cell populations 1 week after the transplantation. Data are shown as the mean ± SD. *n* = 20 (medium; *Col6a1*KO), *n* = 6 (pMSCs), *n* = 10 (iMSCs), and *n* = 6 (KO-iMSCs). *P < 0.05 vs. KO, **P < 0.01 vs. KO, ^§^*P* < 0.05 vs. iMSCs. **d** Sectional images of TA muscle 1 week after medium injection (*COL6a1*KO) or cell transplantation. Yellow arrows indicate MyoD+/Ki67+ nuclei. White arrow heads indicate MyoD+/Ki67- nuclei. Boxed areas indicated in the upper panels are shown with higher magnification in the lower panels. **e** Number of MyoD+ cells 1 week after the transplantation. *n* = 20 (medium; *Col6a1*KO), *n* = 6 (pMSCs), *n* = 10 (iMSCs), and *n* = 6 (KO-iMSCs). **f** % of Ki67+ cells per MyoD+ cells in the TA muscles of *Col6a1*KO mice 1 week after medium injection (*Col6a1*KO) or cell transplantation. *n* = 20 (medium; *Col6a1*KO), *n* = 6 (pMSCs), *n* = 10 (iMSCs), and *n* = 6 (KO-iMSCs). **g** Sectional images of TA muscles 1 week after iMSC transplantation. Yellow arrows indicate Pax7+ cells or MyoD+ cells. Data in **b**,**e**,**f** are shown as the mean ± SD. *P < 0.05. **P < 0.01. n.s., not significant

Subsequently, the effects of COL6 supplementation on MuSC proliferation and differentiation were examined. Previous studies have reported that COL6 acts on quiescent (Pax7+/MyoD-) MuSC self-replication in intact skeletal muscle tissue and increases the number and diameter of regenerating central nuclei fibers during regeneration in response to damage caused by cardiotoxin injection (35). Since COL6 supplementation in intact *Col6a1*KO skeletal muscle enhances regeneration (Figure 4a–e), how COL6 contributes to MuSCs in the uninjured situation was analyzed. In TA muscles transplanted with pMSCs, iMSCs, or KO-iMSCs, the number of Pax7+ MuSCs tended to increase compared to untreated *Col6a1*KO TA muscles but not significantly (Figure 5a, b). The abundance ratio of quiescent (Pax7+/MyoD-) MuSCs and activated (Pax7+/MyoD+) MuSCs was examined. In skeletal muscle supplemented with COL6 by the cell transplantation, the ratio of activated MuSCs increased (Figure 5c). Furthermore, the number of MyoD+ myoblasts increased with either pMSC or iMSC transplantation, but not with KO-iMSC transplantation (Figure 5d, e). More than 60% of MyoD+ cells were Ki67-positive proliferative myoblasts for both the pMSC and iMSC transplantation, but less than 40% for the KO-iMSC transplantation (Figure 5d, f). The increased CSA per eMHC-positive fiber, the activation of MuSCs, and the proliferation of myoblasts all occurred in the areas supplemented with COL6 protein secreted from the transplanted cells (Figure 5 g). Importantly, the benefits of the pMSC and iMSC transplantations were muted if COL6 was not secreted. These results suggest that COL6 supplementation by the engrafted cells promotes muscle regeneration via MuSC activation and myoblast proliferation.

### rh-COL6 does not promote*Col6a1*KO MuSCs proliferation or maturation in vitro

To confirm in vitro the promotion of skeletal muscle regeneration and maturation shown in the MSC transplantation experiments, primary MuSCs were collected from the skeletal muscle tissue of *Col6a1*KO mice and used in culture experiments. First, to verify the direct effect of COL6, MuSCs were cultured in a rh-COL6 protein-coated dish. Since COL1-coated dishes are usually used to cultivate MuSCs, a COL1-coated dish was used for comparison.

The number of cells on day 3 culture was not significantly different between COL1-coated and COL6-coated dishes (Figure S8a, b). Myogenesis progressed similarly, and the ratio of Pax7+/MyoD+ cells was high in both cases (Figure S8c, d).

There was no significant difference in the number of MHC-positive myotubes, the fusion index, or the CSA of MHC-positive myotubes between the two coats on day 6 culture (Figure S8e, f).

### COL6 produced by co-cultured cells promotes the proliferation of *Col6a1*KO-MuSCs and enhanced the maturation of differentiated myotubes

**Fig. 6:**
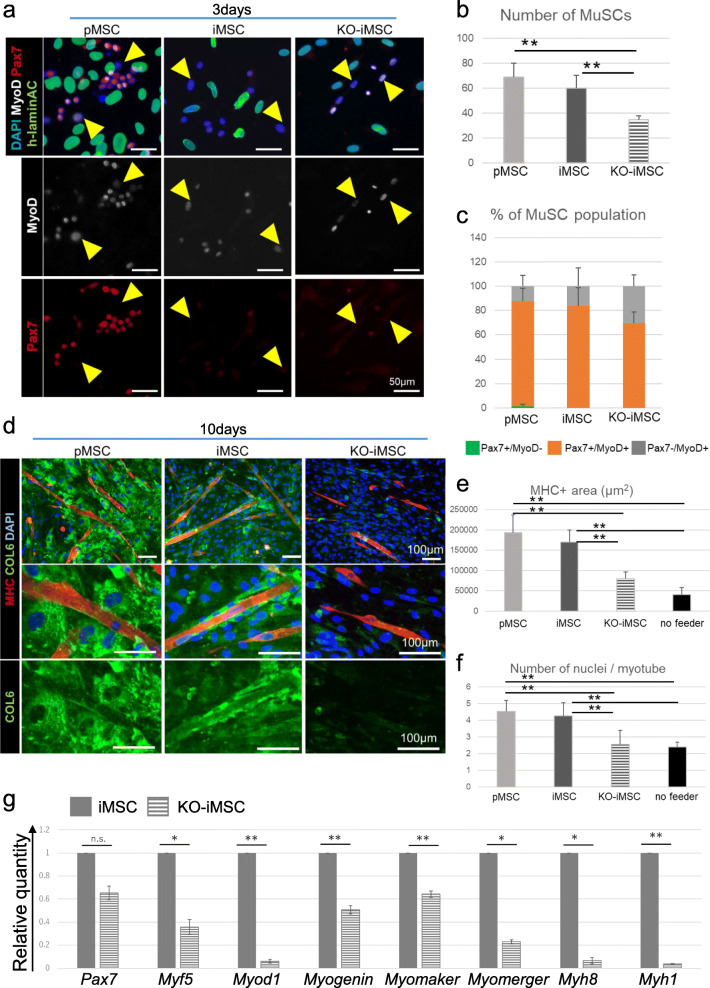
Effect of COL6 protein on myogenesis in co-culture experiments. **a** Immunofluorescence images of *Col6a1*KO mouse-derived MuSCs 3 days after co-culture with pMSCs, iMSCs, or KO-iMSCs. Yellow arrowheads indicate MyoD+/Pax7- cells. **b** Number of DAPI+/human Lamin A/C- mouse MuSCs 3 days after co-culture. Data of three independent experiments are shown as the mean ± SD. **P < 0.01. **c** Percentage of Pax7 + MyoD–, Pax7 + MyoD+ and Pax7–MyoD+ cell populations 3 days after co-culture. Data of three independent experiments are shown as the mean ± SD. **d** Immunofluorescence images of *Col6a1*KO mouse-derived MuSCs 10 days after co-culture with pMSCs, iMSCs, or KO-iMSCs. Nuclei were stained with DAPI. **e** The area of MHC+ myotubes derived from mouse MuSCs 10 days after co-culture. Data of three independent experiments are shown as the mean ± SD. **P < 0.01. **f** Number of nuclei in MHC+ myotubes derived from mouse MuSCs 10 days after co-culture. Data of three independent experiments are shown as the mean ± SD. **P < 0.01. **g** The expression of marker genes. The mRNA expression of each gene was analyzed by RT-qPCR in primary MuSCs derived from *Col6a1*KO mice 6 days after co-culture with iMSCs or KO-iMSCs. Expressions are shown relative to values in MuSCs co-cultured with iMSCs for 6 days. MuSC marker: *Pax7*; myogenic markers: *Myf5*, *Myod1*, and *Myogenin*; myoblast fusion-related genes: *Myomaker* and *Myomerger*; muscle fiber-maturation markers: *Myh8* and *Myh1*. Data of three independent experiments are shown as the mean ± SD. **P < 0.01

Next, *Col6a1*KO-MuSCs were co-cultured with pMSCs, iMSCs, or KO-iMSCs. After culturing in growth medium for 3 days, the number of human Lamin A/C-negative nuclei was counted to verify the expansion of MuSCs. MuSCs proliferated significantly more when co-cultured with either pMSCs or iMSCs than with KO-iMSCs (Figure 6a, b), but the activation status of the MuSCs was similar among the three groups (Figure 6c). In all three cases, however, compared to the case without co-culture with feeder cells, the proportion of Pax7+/MyoD- quiescent satellite cells decreased and the proportion of Pax7-/MyoD+ myoblasts increased (compare Figure 6a, c and Figure S8c, d). These results indicate that the differentiation of satellite cells was promoted by the co-culture.

pMSCs and iMSCs were found near the formed myotubes, and the myotubes appeared to be wrapped with secreted COL6 on day 10 of the co-culture (Figure 6d). Also, MuSCs in these co-cultures showed an expanded MHC positive area, indicating that the differentiation and maturation of skeletal muscle was promoted by COL6 protein secreted by pMSCs and iMSCs (Figure 6d, e). These effects were significantly dampened in KO-iMSC co-cultures. Furthermore, the number of nuclei contained in the myotubes was significantly increased in MHC-positive cells differentiated from MuSCs co-cultured with pMSCs and iMSCs, but not with KO-iMSCs or no co-culture (Figure 6f). These observations indicate that COL6 protein secreted from pMSCs and iMSCs promoted myoblast fusion and myotube maturation. Interestingly, myotubes derived from MuSCs co-cultured with either pMSCs or iMSCs showed sarcomere structures and spontaneous contractions on day 10 of the co-culture (Supplemental movie 1). On the other hand, while MuSCs co-cultured with KO-iMSCs could form myotubes, they did not show sarcomere structures or spontaneous contractions on day 10 (Supplemental movie 2).

Three days after replacing the co-culture with differentiation medium, the expression levels of genes related to skeletal muscle differentiation and maturation were analyzed by qPCR using specific murine gene primers (Figure 6g). As a result, the expressions of all myogenic differentiation markers (*Myf5*, *Myod1*, *Myogenin*), myotube maturation markers (*Myh8*, *Myh1*), and markers related to skeletal myoblast fusion (*Myomaker*, *Myomerger*) were significantly increased in MuSCs co-cultured with iMSCs for 6 days, but the expression level of *Pax7* was low in MuSCs co-cultured with iMSCs or KO-iMSCs with no significant difference (Figure 6g). These results indicate that COL6 secreted from iMSCs promotes myogenic differentiation, myoblast fusion, and myotube maturation.

To validate the potential for myogenesis by COL6, *Col6a1*,*2* knockdown (KD) experiments were conducted in another fibroblastic cell type. Using *MyoD-RFP* mice, myogenic cells were isolated by RFP expression and co-cultured with mouse embryonic fibroblasts (MEFs) that expressed COL6 (Figure S9a). The MEFs were treated with siRNA for *Col6a1* and *Col6a2* (Figure S9b, Table S6). RFP-positive myogenic cells proliferated better when co-cultured with control MEFs than with KD MEFs (Figure S9c). These results indicate that skeletal muscle differentiation and maturation are delayed by *Col6a1*,*2* KD.

### UCMD patient-derived iMSCs showed defective potential for promoting muscle regeneration

Next, a validation experiment using patient-derived iMSCs was conducted. The donor of these patient-derived iMSCs has a missense mutation in the *COL6A1* gene, which creates protein misfolding and prevents the creation of COL6 with the correct tertiary structure in skeletal muscle tissue. As a result, the misfolded COL6 in this patient is unable to function and develops a UCMD pathology. The UCMD-iMSCs expressed MSC markers similar to MSCs and iMSCs (Figure S10). In addition, to confirm that the secreted COL6 protein had a normal three-dimensional structure, immunofluorescence staining was performed using an antibody that recognizes only the triple helical domain of the protein (t-COL6 antibody). iMSCs derived from healthy subjects were stained not only with the pan COL6 antibody but also with t-COL6 antibody (Figure S10b). On the other hand, in UCMD-iMSCs, the COL6 protein was not detected by the t-COL6 antibody, but it was recognized by the pan COL6 antibody. These results indicate that UCMD-iMSCs have the ability to produce COL6 but not with the proper three-dimensional structure.

**Fig. 7:**
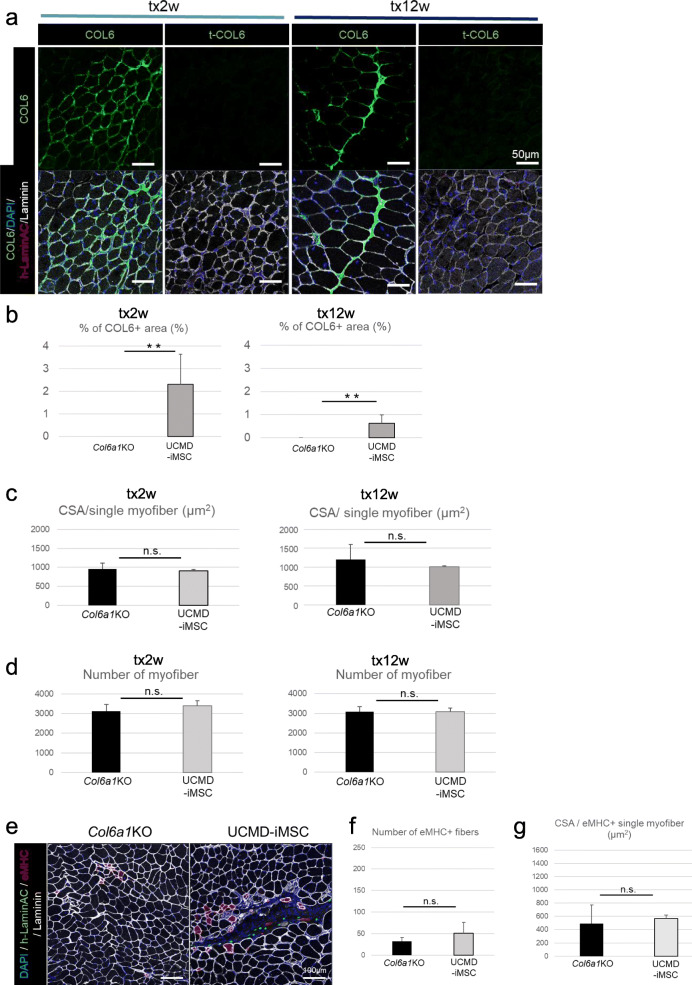
UCMD-iMSC transplantation experiments. **a** Sectional images of TA muscle 2 weeks (tx2w, left 2 columns) and 12 weeks (tx12w, right 2 columns) after UCMD-iMSC transplantation. **b** Quantitative data of the COL6-positive area per whole TA muscle CSA 2 and 12 weeks after the transplantation. Data are shown as the mean ± SD. *n* = 6 (2 weeks), *n* = 6 (12 weeks). **P < 0.01. **c** CSA per muscle fiber in the TA muscles of *Col6a1*KO mice 2 and 12 weeks after medium injection or UCMD-iMSC transplantation. Data are shown as the mean ± SD. *n* = 3. n.s., not significant. **d** Number of muscle fibers in the TA muscles of *Col6a1*KO mice 2 and 12 weeks after medium injection or UCMD-iMSC transplantation. Data are shown as the mean ± SD. *n* = 3. n.s., not significant. **e** Sectional images of TA muscles 1 week after medium injection or UCMD-iMSC transplantation. **f** Number of eMHC-positive fibers 1 week after medium injection or UCMD-iMSC transplantation. Data are shown as the mean ± SD. *n* = 3. n.s., not significant. **g** CSA per eMHC+ muscle fiber in the TA muscles of *Col6a1*KO mice 1 week after medium injection or UCMD-iMSC transplantation. Data are shown as the mean ± SD. *n* = 3. n.s., not significant

Then, UCMD-iMSCs were transplanted into *Col6a1*KO mice. Two and 12 weeks after the transplantation, the secretion of COL6 protein around the transplanted cells was detected by the pan COL6 antibody but not by the t-COL6 antibody (Figure 7a). COL6 was secreted and expanded at almost the same level as that seen in the iMSC transplantation experiments (Figure 7b). The CSA and number of muscle fibers were measured in the muscle tissue samples 2 and 12 weeks after the transplantation. No significant difference was detected between the control limb (no transplantation) and the UCMD-iMSC transplanted limb (Figure 7c, d).

Further analysis of eMHC-positive regenerative muscle fibers in the tissue sample 1 week after the transplantation revealed no hypertrophy (Figure 7e). Again, no significant difference was detected between the control limb and the UCMD-iMSC transplanted limb (Figure 7f, g). These results indicated that COL6 produced by iMSCs derived from dominant negative type UCMD patient cells had no therapeutic effect on *Col6a1*KO mouse muscle.

## Discussion

In the present study, iMSCs promoted the regeneration and maturation of skeletal muscles in *Col6a1*KO UCMD model mice in vitro and in vivo. On the other hand, iMSCs derived from two types of COL6-deficient iPSCs (KO-iMSCs and UCMD-iMSCs) had no effect on improving the phenotype. Overall, these results demonstrated that MSC therapies depend on the secretion of COL6 for their therapeutic effect on UCMD model mice.

The pathogenesis mechanism of COL6-related diseases has been roughly classified into the following: autophagy abnormality (31–33), apoptosis enhancement due to mitochondrial dysfunction (25–30), and skeletal muscle regeneration/maturation deficiency (34, 35). The current study focused on the mechanism for skeletal muscle regeneration/maturation deficiency in UCMD and the development of new therapeutic methods.

Skeletal muscle regeneration and maturation deficiency has been reported as a characteristic phenotype in muscle biopsy samples of human UCMD patients (36) and UCMD model mice (40, 41). However, little is known about the COL6-dependent mechanism for the occurrence and progression of the deficiency. Previous reports about cell transplantation treatment in UCMD model mice have only given short-term observations (34, 35), and no mention has been made as to whether the therapeutic effect of the transplantation is temporary or long-lasting. In the present study, the course up to 24 weeks after the transplantation was followed. At 12 weeks, no benefit was seen with COL6-deficient iMSCs, but significant muscle fiber hypertrophy was induced with COL6-producing pMSCs/iMSCs. These results suggest a long-term effect that promotes muscle maturation. The transplanted pMSCs/iMSCs and secreted COL6 were still present at 24 weeks, and the therapeutic effect was maintained.

To directly demonstrate that the pathological phenotype depended on COL6, two types of COL6-deficient MSCs: KO-iMSCs and UCMD-iMSCs were created. Previous studies have reported that factors secreted from healthy MSCs can promote muscle regeneration (myogenesis)(42, 43). On the other hand, MSCs in UCMD muscle may have reduced or lost this muscle regeneration ability(40). Thus, MSC supplementation, regardless of COL6 expression, may temporarily improve the phenotype. In the present study, the transplantation of KO-iMSCs slightly activated myoblasts, but far less so than when pMSCs/iMSCs were transplanted. Moreover, COL6 supplementation by the transplantation of pMSCs/iMSCs significantly promoted the number of nuclei in eMHC-positive regenerating myofibers and the CSA of the myofibers, but these observations not made with KO-iMSC transplantation. This finding is consistent with the co-culture experiments in this study: co-culture with iMSCs promoted the growth, fusion, and maturation of *Col6a1*KO-MuSCs, and the expression of genes related to MuSC activation and myotube fusion increased, but not when KO-iMSCs were used for the co-culture. These results indicate that the effect of MSC transplantation on myoblast fusion and muscle fiber maturation is synergized by COL6 secretion.

The in vitro experiments in this study showed that iMSC co-culture promoted myoblast proliferation and muscle maturation, consistent with the in vivo experiments. Considering that this effect was not observed when using COL6-coated dishes, we hypothesized that the COL6 effect requires three-dimensional interactions of the protein with the surrounding myoblasts and myofibers. Consistently, transplanted pMSCs/iMSCs tightly surrounded and wrapped the myotubes with secreted COL6 protein. As for why rh-COL6 did not have this effect, one possibility is that the protein cannot diffuse, thus remaining in only a small portion of the injected muscle and vanishing in less than 2 weeks. Without COL6-producing cells (i.e., pMSCs/iMSCs), sufficient COL6 protein may not be delivered and sustained in UCMD muscle.

There have been two previous reports on cell transplantation for UCMD. The primary cells used in those studies showed a certain therapeutic effect on UCMD muscles (34, 35). However, the practical application of primary cell-based therapy is difficult, because primary cells have limited growth (52). In fact, the pMSCs derived from adult skeletal muscle tissue used in this study as a positive control have an upper limit for subculture in vitro, and changes in their properties such as differentiation and proliferation ability occurred after about 3–5 passages (data not shown). Furthermore, tissue collection from donors always involves invasion, and the variability in properties between donors is large (53, 54). Finally, primary cells from patients have mutations in *COL6A1-3*, such that autologous transplants cannot be used therapeutically. In contrast, iPSCs, which are the source of iMSCs, can be propagated indefinitely while maintaining stemness. Especially, iMSCs created from HLA-edited iPSCs may be a universal therapeutic product (55).

This study did not confirm the improvement of muscle motor function by iMSC transplantation. However, in UCMD model mouse neonates transplanted iMSCs intraperitoneally and systemically, COL6 protein was expressed in skeletal muscle tissue of the whole body and could improve motor function (Harada et al., in revision). Therefore, there is a high possibility that iMSCs can be used as a cell source for transplantation in place of pMSCs.

## Conclusions

Two main key findings were demonstrated in this study. First is that iMSCs have equivalent therapeutic effects to pMSCs in UCMD model mice, such as the enhancement of muscle regeneration and maturation. Second is that these therapeutic effects are mediated by COL6 supplementation. In the future, it is expected that iMSCs derived from HLA-edited iPSCs will be used for clinical application in patients. These results not only raise the possibility of cell transplantation treatment as a new therapeutic means but could also help to select treatment targets when developing new therapeutics for UCMD.

## Supplementary Information

**Additional file 1:** Supplemental Tables. Tables S1-S6

**Additional file 2:** Supplemental Methods

**Additional file 3:** Supplemental Figures and Legends. Figures S1-S10

**Additional file 4:** Supplemental movie 1

**Additional file 5:** Supplemental movie 2

## Data Availability

All raw data files for the RNA-sequencing are available to the public through the DDBJ submission portal D-way (DRA009935/ftp://ftp.ddbj.nig.ac.jp/ddbj_database/dra/fastq/DRA009/DRA009935/). KO-iPSC and UCMD-iPSC will be deposited in the RIKEN BRC.

## Abbreviations

COL6: Type 6 collagen
MSCs: Mesenchymal stromal cells
UCMD: Ullrich congenital muscular dystrophy
iPSC: Induced pluripotent stem cell
iMSCs: iPSC-derived MSCs
MuSCs: Skeletal muscle satellite cells
ECM: Extracellular matrix
KO-iPSCs: *COL6A1* knocked out iPSC
UCMD-iPSCs: iPSCs generated from UCMD patients
KO-iMSCs: KO-iPSC-derived MSCs
UCMD-iMSCs: UCMD-iPSC-derived MSCs
pMSCs: Primary MSCs derived from human skeletal muscle tissues
NSG mouse; severe immunodeficient: NOD.Cg-Prkdc^scid^Il2rg^tm1Wjl^/SzJ
*Col6a1*KO mouse: *Col6a1*^GT/GT^/NSG mouse
NCCs: Neural crest cells
TA muscle: Tibialis anterior muscle
rh-COL6: Recombinant human COL6
CSA: Cross-sectional area
