# Supplementary material for: Collagen-VI supplementation by cell transplantation improves muscle regeneration in Ullrich congenital muscular dystrophy model mice
Source: Stem Cell Res Ther. 2021 Aug 9;12:446. doi: 10.1186/s13287-021-02514-3 (PMC8351132; doi:10.1186/s13287-021-02514-3)
Supplement: Supplementary file 1 — Additional file 1: Supplemental Tables. Tables S1-S6 [file 13287_2021_2514_MOESM1_ESM.docx]

**Supplemental Tables**

| **Table S1. List of primers and template genes used for construction of plasmids** | | |
| --- | --- | --- |
| Part of Fragment |  | Sequence |
| *For Reporter Plasmids* | | |
| 5' Arm | Fw | GGAACCAATTCAGTCGACTGCACTCTGGCTGGGAGCAGAAGGCAGC |
|  | Rv | CGAAGTTATGAATTCCGGAGTGGTGAAGGACTTGACTTTGTCCACGAGGG |
|  | Temp | Human Genome |
| 3’ Arm | Fw | AGTTATACTAGTTTAAACGCGACAGGTAGGAGGGACGCCCCGTGAC |
|  | Rv | GGTCTAGATATCTCGAGTGCGAAACTGAGTCATGGGGAGACTGAGTAAAG |
|  | Temp | Human Genome |
| *For sgRNA Plasmids* | | |
| sgRNA | Fw | GAGACCACTTGGATCCAGCGCTTCATCGACAACCTGGTTTTAGAGCTAGAAATAGCA |
|  | Rv | GCCCGGGTTTGAATTCAAAAAAAGCACCGACTCGGTGCCACTTTTTCAAGTTGATAACGGACTAGCCTTATTTTAACTTGCTATTTCTAGCTC |
|  | Temp | - |
| **Table S2. List of primers used for genotyping and copy number testing** | | |
| *Genotyping* | | |
| Geno-  typing | Fw | TTGTGAGCAACTTTGTCTTCCCACGGTA |
|  | Rv | ACACCAGGTTTCGGTCACAGCGGTAGTA |
| *Copy Number Testing* | | |
| Puro | Fw | CGCTCGTAGAAGGGGAGGTT |
|  | Rv | CACCAGGGCAAGGGTCTG |
| DLX5 | Fw | CCCCGTAGGGCTGTAGTAGT |
|  | Rv | TTCCAAGCTCCGTTCCAGAC |

**Table S3. List of antibodies used**

For immunocytochemistry and immunohistochemistry

| **Antigen** | **Host species** | **Clone** | **Conjugate** | **Source** | **Cat. No.** | **Dilution** |
| --- | --- | --- | --- | --- | --- | --- |
| COL6 (pan) | rabbit | poly | - | abcam | ab6588 | 1/200 |
| mouse-PDGFRα | goat | poly | - | R&D | AF1062 | 1/15 |
| human-PDGFRα | goat | poly | - | R&D | AF-307-NA | 1/20 |
| Laminin-α2 | rat | mono | - | ALEXIS | ALX-804-190-C100 | 1/50 |
| human-Lamin A/C | mouse | mono | - | Santacrutz | sc-7292 | 1/200 |
| eMHC (MYH3) | rabbit | poly | - | Sigma | HPA021808 | 1/200 |
| human nuclei | mouse | mono | - | Merck | MAB1281 | 1/200 |
| Pax7 | mouse | mono | - | DSHB | AB_528428 | 1/100 |
| MyoD | rabbit | mono | - | abcam | ab133627 | 1/500 |
| Ki67 | mouse | mono | Alexa647 | BD | 558615 | 1/500 |
| MHC (Myosin heavy chain) | mouse | mono | - | eBioscience | 14-6503-82 | 1/800 |
| t-COL6  (triple helical domain) | mouse | mono | - | Kyowa ohama chemical | F-62 | 1/50 |
| RFP | rabbit | poly | - | MBL | PM005 | 1/1000 |
| TnT (Troponin T) | mouse | mono | - | Sigma | T6277 | 1/200 |
| Rabbit IgG | donkey | poly | Alexa488 | Thermo Fisher Scientific | R37118 | 1/500 |
| Goat IgG | donkey | poly | Alexa568 | Thermo Fisher Scientific | A11057 | 1/500 |
| Rat IgG | donkey | poly | Alexa647 | abcam | ab150155 | 1/500 |
| Mouse IgG | donkey | poly | Alexa488 | Thermo Fisher Scientific | R37114 | 1/500 |
| Rabbit IgG | goat | poly | Alexa488 | Thermo Fisher Scientific | A11034 | 1/500 |
| Rabbit IgG | goat | poly | Alexa568 | Thermo Fisher Scientific | A11036 | 1/500 |
| Rabbit IgG | goat | poly | Alexa647 | Thermo Fisher Scientific | A27040 | 1/500 |
| Rat IgG | goat | poly | Alexa647 | Thermo Fisher Scientific | A21247 | 1/500 |
| Mouse IgG1 | goat | poly | Alexa568 | Thermo Fisher Scientific | A21124 | 1/500 |
| Mouse IgG1 | goat | poly | Alexa488 | Thermo Fisher Scientific | A21121 | 1/500 |
| Mouse IgG2b | goat | poly | Alexa568 | Thermo Fisher Scientific | A21144 | 1/500 |
| Mouse IgG2b | goat | poly | Alexa488 | Thermo Fisher Scientific | A21141 | 1/500 |

For flow cytometry

| **Antigen** | **Host species** | **Clone** | **Conjugate** | **Source** | **Cat. No.** | **Dilution** |
| --- | --- | --- | --- | --- | --- | --- |
| CD45 | Rat | mono | PE | BioLegend | 103106 | 1/200 |
| CD31 | Rat | mono | PE | BioLegend | 102508 | 1/200 |
| Sca-1 | Rat | mono | PE | BioLegend | 122508 | 1/200 |
| Streptavidin | - | - | APC | BD Pharmingen | 349024 | 1/400 |
| CD201 | rat | mono | PE | BioLegend | 351904 | 1/40 |
| CD44 | mouse | mono | APC | BD Pharmingen | 559942 | 1/100 |
| CD45 | mouse | mono | APC | BD Pharmingen | 560973 | 1/100 |
| CD73 | mouse | mono | PE | BD Pharmingen | 550257 | 1/100 |
| CD105 | mouse | mono | APC | eBioscience | 17-1057 | 1/100 |
| p75 (CD271) | mouse | mono | Alexa647 | BD Pharmingen | 560326 | 1/100 |
| CD56 | mouse | mono | VioBright FITC | Miltenyi Biotec | 130-104-944 | 1/20 |
| CD56 | mouse | mono | PE- | Miltenyi Biotec | 130-090-755 | 1/20 |
| CD56 | mouse | mono | PE/Cy7 | BD Pharmingen | 335791 | 1/40 |
| PDGFRa (biotinylated) | goat | poly | - | R&D | BAF322 | 1/80 |
| PDGFRa | mouse | mono | BB515 | BD Pharmingen | 564594 | 1/40 |
| Streptavidin | - | - | PE/Cy5 | BD Pharmingen | 554062 | 1/200 |
| Goat IgG | donkey | poly | PE | R&D | 705-116-147 | 1/200 |
| Mouse_IgG2b_κ | mouse | mono | PE | BioLegend | 400312 | 1/100 |
| Mouse_IgG1_κ | mouse | mono | PE | BioLegend | 400112 | 1/100 |
| Mouse_IgG1_κ | mouse | mono | APC | BioLegend | 400120 | 1/100 |
| Rat_IgG1_κ | rat | mono | PE | BioLegend | 400408 | 1/100 |

For western blotting

| **Antigen** | **Host species** | **Clone** | **Conjugate** | **Source** | **Cat. No.** | **Dilution** |
| --- | --- | --- | --- | --- | --- | --- |
| COL6 | rabbit | poly | - | abcam | Ab199720 | 1/5000 |
| b-Actin | mouse | mono | HRP | Sigma | A3854 | 1/40000 |
| GAPDH | mouse | mono | - | Millipore | MAB374 | 1/5000 |
| Rabbit IgG (biotinylated) | goat | poly | - | VECTOR | BA1000 | 1/500 |
| Streptavidin | - | - | HRP | BioLegend | 405210 | 1/4000 |
| Mouse IgG | goat | poly | HRP | abcam | Ab98693 | 1/20000 |

**Table S4. List of mouse genotyping primers used**

| strain name |  | sequences |
| --- | --- | --- |
| *Col6a1*KO | Fw | TTCCAGAGCAGATGGCTGTGGCTC |
|  | wild type Rv | ACCCAGGATGGAGGAGAGGTTATG |
|  | mutant Rv | GCTAGACTAGTACGCGTGTACACT |
| NSG | Fw | GTGGGTAGCCAGCTCTTC AG |
|  | wild type Rv | CCTGGAGCTGGACAACAA AT |
|  | KO Rv | GCCAGAGGCCACTTGTGTAG |

**Table S5. List of qPCR primers used**

| gene | Fw | Rv |
| --- | --- | --- |
| human *NANOG* | CAGTCTGGACACTGGCTGAA | CTCGCTGATTAGGCTCCAAC |
| human *SOX2* | GGGAAATGGGAGGGGTGCAAAAGAGG | TTGCGTGAGTGTGGATGGGATTGGTG |
| human *OCT3/4* | GACAGGGGGAGGGGAGGAGCTAGG | CTTCCCTCCAACCAGTTGCCCCAAAC |
| human *COL6A1* | Ctcctctgtccccatagctg | ctgaccttggtttcccaaaa |
| human *PDGFR-A* | ggccccatttacatcatcac | catagctccgtgtgctttca |
| human *ACTB* | CTCTTCCAGCCTTCCTTCC | CACCTTCACCGTTCCAGTTT |
| mouse *Pax7* | AGGCCTTCGAGAGGACCCAC | CTGAACCAGACCTGGACGCG |
| mouse *Myf5* | GTCTACAGAGCCATCCGAGC | GCAGGAGTGATCATCGGGAG |
| mouse *Myod1* | AGCACTACAGTGGCGACTCA | GGCCGCTGTAATCCATCAT |
| mouse *Myogenin* | CAACCAGGAGGAGCGCGATCTCCG | GGCGCTGTGGGAGTTGCATTCACT |
| mouse *Myomaker* | ATCGCTACCAAGAGGCGTT | CACAGCACAGACAAACCAGG |
| mouse *Myomerger-S* | CAGGAGGGCAAGAAGTTCAG | ATGTCTTGGGAGCTCAGTCG |
| mouse *Myh8* | AGGTTCACACCAAAATCAGCG | CCCTCCTGTGCTTTCCTTCAG |
| mouse *Myh1* | CATCCCTAAAGGCAGGCTCT | AGCCTCGATTCGCTCCTTTT |
| mouse *Col6a1* | CTGCTGCTACAAGCCTGCT | CCCCATAAGGTTTCAGCCTCA |
| mouse *Col6a2* | AAGGCCCCATTGGATTCCC | CTCCCTTCCGACCATCCGAT |
| mouse *Gapdh* | TGCGACTTCAACAGCAACTC | GCCTCTCTTGCTCAGTGTCC |
| mouse *Rpl13a* | GTGGTCCCTGCTGCTCTCAAG | CGATAGTGCATCTTGGCCTTTT |

**Table S6. List of siRNA used**

| siRNA-*Col6a1* | Sigma | mission siRNA Col6a1 4407 |
| --- | --- | --- |
| siRNA-*Col6a2* | Sigma | mission siRNA Col6a2 1549 |
