# Supplementary material for: Collagen-VI supplementation by cell transplantation improves muscle regeneration in Ullrich congenital muscular dystrophy model mice
Source: Stem Cell Res Ther. 2021 Aug 9;12:446. doi: 10.1186/s13287-021-02514-3 (PMC8351132; doi:10.1186/s13287-021-02514-3)
Supplement: Supplementary file 2 — Additional file 2: Supplemental Methods [file 13287_2021_2514_MOESM2_ESM.docx]

**Supplemental Methods**

**Generation of iPSCs**

The human iPSC line (201B7) from a healthy donor was used([1](#_ENREF_1)). UCMD patient-derived iPSCs were established from the dermal fibroblasts of a UCMD patient who has a mutation in *COL6A1* (c.860C>A) according to the methods reported in a previous study([2](#_ENREF_2)). All iPSCs were cultured and maintained in feeder-free culture on iMatrix-511 (Nippi, Tokyo, Japan) in StemFit AK02N medium (Ajinomoto, Tokyo, Japan) as previously described([3](#_ENREF_3))**.**

**Isolation of MuSCs from human**

Non-dystrophic healthy muscle samples were obtained from the gluteus medius muscles of female subjects undergoing total hip arthroplasty. The methods for dissociating cells from the muscle samples and sorting MuSCs (CD56+) are well described in a previous report([4](#_ENREF_4)). The procedure used for the isolation, culture and maintenance is the same as that used for pMSCs described in the main text.

**Isolation of MuSCs and myoblasts from mice**

MuSCs were isolated from 4-6-week-old male *Col6a1*KO mice. The skeletal muscles in the hind legs were collected and digested into single cells with a Satellite Cell Isolation Kit (Miltenyi Biotec, Bergisch Gladbach, Germany) according to the manufacturer’s protocol. The isolated cells were stained with PE-conjugated anti-CD45, anti-CD31, anti-Sca-1, and biotinylated SM/C-2.6 antibodies([5](#_ENREF_5)) (Table S3).  The cells were then incubated with streptavidin-APC on ice for 30 minutes and resuspended in HBSS containing Hoechst (1:2000). Cell sorting was performed using a FACS Aria II flow cytometer, and the data obtained were analyzed using FACS Diva software. The sorted cells were counted and seeded (5 x 10^3^/well) on type 1 collagen-coated 24-well plates (Iwaki, Shizuoka, Japan) in MuSC expansion medium.

E14 (embryonic day 14) MyoD1-positive myoblasts were sorted from male and female MyoD-RFP mouse embryos according to a method described previously([6](#_ENREF_6), [7](#_ENREF_7)). Similarly, MEFs (mouse embryonic fibroblasts) were collected and used for the *in vitro* co-culture experiments([6](#_ENREF_6), [7](#_ENREF_7)).

**Generation of *COL6A1*KO iPSC line**

To construct the sgRNA expression vector, oligos containing the sgRNA 5’-AGCGCTTCATCGACAACCTG-3’ and a universal reverse primer were PCR amplified and cloned into the BamHI-EcoRI site of the pHL-H1-ccdB-mEF1α-RiH vector. All primers are listed in Table S1. LoxP-puro-LoxP was inserted into pENTR1A vectors using Gateway pENTR^TM^ vectors (A10462, Thermo Fisher Scientific, Waltham, MA, USA) to construct the pENTR-LoxP-puro-LoxP vector (pENTR-Donor-MCS2). The fragments *COL6A1*-5’Arm and *COL6A1*-3’Arm were cloned by PCR (KOD Plus Neo, TOYOBO, Osaka, Japan). All primers used in the PCR are listed in Table S1. Each fragment was integrated into the BamH1 or Not1 site of the pENTR-LoxP-puro-LoxP vector using NEBuilder HiFi DNA Assembly Master Mix (E2621, New England Biolabs (NEB), Ipswich, MA, USA). Before the transfection experiments, plasmid DNAs were purified using the NucleoBond Xtra Maxi Plasmid DNA Purification Kit (U0412B, Takara Bio, Shiga, Japan).

Together with the Cas9 expression vector (pHL-EF1α-SphcCas9-iP), the donor template vector and the sgRNA expression vector were electroporated into 201B7 iPSCs as previously described([8](#_ENREF_8)). Briefly, transfection was performed with an NEPA 21 Electroporator (Nepa gene, Chiba, Japan) as follows. The poring pulse was set to pulse voltage, 125 V; pulse width, 5 ms; and pulse number, 2. Human iPSCs were pretreated with a ROCK inhibitor (10 µM Y-27632; 08945-71, Sigma-Aldrich, St. Louis, MO, USA) for at least 1 h before electroporation and dissociated into single cells with 0.25% Trypsin solution treatment for 5 min at 37°C. 5 µg of Cas9 plasmid, 5 µg of sgRNA plasmid, and 5 µg of donor template plasmid were electroporated into 1 x 10^6^ cells. After the electroporation, the cells were plated onto an iMatrix-511-coated 10 cm dish in the presence of 10 µM Y-27632 for 2 days.

48 h after the electroporation, the iPSCs were treated with 1 µg/mL puromycin for the selection of transfected cells. After subcloning the 24 colonies, genome DNA was extracted from each clone for genotyping. To select homozygous *COL6A1*-puro cell lines, genomic PCR was done by genotyping Fw1-Rv1 primer pairs, then the copy numbers of the knocked-in construct were checked by qRT-PCR (Table S2). After confirmation, one homozygous clone was selected and transfected with 5 µg CRE expression vector pCMV-Cre-puro to remove the LoxP-puro-LoxP cassette. After transfection, we picked up several clones and sequenced each genome in order to confirm the stop codon was correctly knocked in.

**Immunocytochemistry and immunohistochemistry**

Prior to performing immunostaining, cells on plates and muscle tissue sections on slides were fixed with 2% (cells) and 4% (muscle tissue sections) PFA/PBS (FUJIFILM Wako, Osaka, Japan) at room temperature for 15 min, washed two times with PBS, and incubated with Blocking One (nacalai tesque, Kyoto, Japan) at room temperature for 1 h to block any nonspecific binding. Then the primary antibody, which was diluted with Can Get Signal Solution B (NKB-601, TOYOBO), was added to the sample, and the sample was washed two times with PBS and Triton X100 (nacalai tesque). Finally, the secondary antibody, DAPI (1:1000; Thermo Fisher Scientific), which was used to counterstain nuclei, and Can Get Signal Solution B were added to the sample, and the sample was washed two times with PBS and Triton X100. The primary and secondary antibodies used are summarized in Table S3. Observations and assessments of the samples were performed with a BZ-X700 (Keyence, Osaka, Japan).

**FACS analysis**

The cells were trypsinized, re-suspended in HBSS, and stained with primary antibodies for 30 min at 4°C. The primary antibodies and isotype controls used are summarized in Table S3. Stained cells were analyzed with FACS LSR (BD Biosciences, San Jose, CA, USA) and FlowJo_v10.6.1.

**RNA-seq analysis**

Total RNA was obtained from pMSCs, iMSCs, KO-iMSCs, primary MuSCs, and undifferentiated iPSCs. The sample qualities were evaluated using a NanoDrop 2000 (Thermo Fisher Scientific) and Agilent 2100 Bioanalyzer System (Agilent, Santa Clara, CA, USA) with the RNA Pico Kit (Agilent 5067-1513). Index-labeled cDNA libraries were prepared from the RNA samples using the TruSeq Stranded mRNA LT Sample Prep Kit (RS-122-2101, Illumina, Inc., San Diego, CA, USA). The libraries were quantified using the Agilent 2100 Bioanalyzer System with High Sensitivity DNA Kit (Agilent, 5067-4626), and equal amount of molecular pools and a NextSeq 500 (Illumina, Inc.) were used for the RNA seq analysis according to the manufacturer’s protocol (1x75bp single read). The raw sequence data were converted to fastq format files. The sequenced reads were trimmed to remove adaptors and low quality bases using the FASTX Toolkit, ver. 0.0.14. Trimmed data were mapped to reference human genome GRCh37 downloaded from NCBI RefSeq using TopHat2, ver. 2.1.1([9](#_ENREF_9)). HTSeq, ver. 0.12.4([10](#_ENREF_10)), was used to calculate gene read counts, and the counted data were normalized by DEseq2, ver. 3.11([11](#_ENREF_11)). Principal component analysis (PCA) and heatmaps were done using R, ver. 3.6.1 (https://cran.r-project.org/bin/macosx/)([12](#_ENREF_12)), and TCC-GUI (https://github.com/swsoyee/TCC-GUI)([13](#_ENREF_13)).

**Co-culture with MuSCs derived from *Col6a1*KO mice**

One day before the MuSC seeding, iMSCs, KO-iMSCs, and pMSCs were seeded (5 x 10^4^/well) on type 1 collagen-coated 24-well plates and cultured in optimal expansion medium for each cell type as described in the main manuscript. 3 h before the seeding of MuSCs, mytomycin C (Kyowa Kirin, Tokyo, Japan) was added to stop the proliferation of MSCs, 2 h later the dish was washed two times with PBS, and MuSC expansion medium (DMEM (Sigma-Aldrich) supplemented with 10% fetal bovine serum (FBS) (556-33865, FUJIFILM Wako) and 2% Ultroser G (15950-017, PALL, Port Washington, NY, USA)) was added. MuSCs derived from *Col6a1*KO mice were seeded (5 x 10^3^/well) on the plate with feeder cells (iMSCs, KO-iMSCs, or pMSCs). After 3 days, the medium was changed to MuSC differentiation medium (DMEM supplemented with 10% FBS). 3, 6, and 10 days after the seeding of MuSCs, the cells were fixed with 2% PFA, immunofluorescence staining was performed, and the myogenesis of MuSCs was evaluated using a KEYENCE BZ-X analyzer (Keyence). An index of myocyte fusion (the fusion index) was made by counting the number of nuclei inside MHC-positive myotubes. This index is often used as an indicator of muscle maturity. 6 days after the MuSC seeding (3 days after changing the medium), all cells including feeder MSCs were collected and analyzed for mRNA expression with mouse specific primers (Table S5).

**Quantitative RT-PCR**

Total RNA was extracted using the ReliaPrep RNA Miniprep System (Z6012, Promega, Madison, WI, USA). cDNA was synthesized from the extracted RNA using a ReverTra Ace qPCR RT kit (FSQ-101, TOYOBO). Quantitative PCR with the SYBR Green system (Applied Biosystems, Foster City, CA, USA) was performed using the Step One Plus Real-Time PCR System (Applied Biosystems) in triplicate. The primer sequences are listed in Table S5.

**rh-COL6 protein injection into *Col6a1*KO mice**

Recombinant human COL6 (rh-COL6) (354261, Corning, Corning, NY, USA ) (50 µL (0.69 mg/mL) was injected into the center of each TA muscle using a 27 G micro-syringe (Myjector syringe; Terumo, Tokyo, Japan).

**Tissue preparation and histological analysis**

Mice were sacrificed at 1, 2, 4, 8, 12 or 24 weeks post cell transplantation. The TA muscles were mounted in Tragacanth Gum (FUJIFILM Wako) and frozen with liquid nitrogen([14](#_ENREF_14)). Three cryosections (10 µm) were obtained with a Cryostat (Leica Biosystems, Nussloch, Germany) from the central portion, 1 mm above the central portion, and 1 mm below the central portion of one TA muscle and used for the stain analysis. The maximum value was adopted as the experimental data. The selected sections were immunofluorescently stained and mounted with VECTASHIELD mounting medium for fluorescence with DAPI (H-1200, VECTOR LABORATORIES, Burlingame, CA, USA). This mounting medium does not cure, therefore, the coverslip can be removed by immersing the slides in PBS. Immunofluorescence images were acquired using a Zeiss LSM 700 laser scanning confocal microscope (Carl-Zeiss, Oberkochen, Germany) and BZ-X 700. The area measurement and cell counting were performed using BZ-H3C software (Keyence). The slides were then immersed in PBS to remove the mounted coverslip, subjected to H&E (hematoxylin and eosin) staining and observed using an Olympus BX51 (Olympus, Tokyo, Japan) and BZ-X 700.

**Coating with rh-COL6 protein**

rh-COL6 was diluted according to a customized method and used to coat type 1 collagen-coated 24-well plates (Iwaki) at 37°C overnight and a concentration of 5 µg/cm^2^. Before seeding the cells, the COL6 solution for the coating was aspirated off, the plate was washed with PBS, and the appropriate medium was added.

**Protein extraction and western blotting analysis**

Cells and muscle tissues were lysed in radio-immunoprecipitation assay (RIPA) buffer (08714-04, nacalai tesque) with protease inhibitor cocktail (25955-11, nacalai tesque) by thorough sonication (UCD-250, Bioruptor). 7-8 µg of protein from cells or 30 µg of protein from tissues were mixed with reducing agent (NP0004, Invitrogen, Carlsbad, CA, USA) and loaded onto 4-12% Bolt Bis-Tris Plus Gels (NM04122BOX, Thermo Fisher Scientific). Bolt Bis-Tris Plus Gels were ran in an Invitrogen protein electrophoresis system for protein separation. The fractionated proteins were transferred to a PVDF membrane using the iBlot system (IB 401002 iBlot Transfer Stack, Thermo Fisher Scientific) with the P0 program. The membrane was blocked with Blocking One (nacalai tesque) and incubated with primary antibody, which was diluted with Can Get Signal Solution 1 (NKB-201, TOYOBO) at 4°C overnight or for 1 h at room temperature. After 3 washes with TBS with 0.05% TWEEN 20 (P7949, Sigma-Aldrich), the membrane was incubated with secondary antibody, which was diluted with Can Get Signal Solution 2 (NKB-101, TOYOBO), for 1 hat room temperature. If necessary, a third antibody diluted with PBS was reacted for 5 minutes at 4°C after 3 more washes with 0.05% TBS-T (see Table S3 for detailed information about the antibodies). Detection was carried out with SuperSignal West Femto Maximum Sensitivity Substrate (34094, Thermo Fisher Scientific). Visualization and semi-quantification of the images were performed using the Image Quant LAS4000 imaging system (GE healthcare, Chicago, IL, USA).

**Supplemental Information**

**Supplemental movie 1 Muscle contraction of *Col6a1*KO-MuSCs co-cultured with iMSCs**

**Supplemental movie 2 Muscle contraction of *Col6a1*KO-MuSCs co-cultured with KO-iMSCs**

**Supplemental References**

1. Takahashi K, Tanabe K, Ohnuki M, Narita M, Ichisaka T, Tomoda K, et al. Induction of pluripotent stem cells from adult human fibroblasts by defined factors. Cell. 2007;131(5):861-72.

2. Okita K, Matsumura Y, Sato Y, Okada A, Morizane A, Okamoto S, et al. A more efficient method to generate integration-free human iPS cells. Nat Methods. 2011;8(5):409-12.

3. Nakagawa M, Taniguchi Y, Senda S, Takizawa N, Ichisaka T, Asano K, et al. A novel efficient feeder-free culture system for the derivation of human induced pluripotent stem cells. Sci Rep. 2014;4:3594.

4. Uezumi A, Nakatani M, Ikemoto-Uezumi M, Yamamoto N, Morita M, Yamaguchi A, et al. Cell-Surface Protein Profiling Identifies Distinctive Markers of Progenitor Cells in Human Skeletal Muscle. Stem Cell Reports. 2016;7(2):263-78.

5. Fukada S, Higuchi S, Segawa M, Koda K, Yamamoto Y, Tsujikawa K, et al. Purification and cell-surface marker characterization of quiescent satellite cells from murine skeletal muscle by a novel monoclonal antibody. Exp Cell Res. 2004;296(2):245-55.

6. Sakai H, Sato T, Sakurai H, Yamamoto T, Hanaoka K, Montarras D, et al. Fetal skeletal muscle progenitors have regenerative capacity after intramuscular engraftment in dystrophin deficient mice. PLoS One. 2013;8(5):e63016.

7. Sato T, Yamamoto T, Sehara-Fujisawa A. miR-195/497 induce postnatal quiescence of skeletal muscle stem cells. Nat Commun. 2014;5:4597.

8. Li HL, Fujimoto N, Sasakawa N, Shirai S, Ohkame T, Sakuma T, et al. Precise correction of the dystrophin gene in duchenne muscular dystrophy patient induced pluripotent stem cells by TALEN and CRISPR-Cas9. Stem Cell Reports. 2015;4(1):143-54.

9. Kim D, Pertea G, Trapnell C, Pimentel H, Kelley R, Salzberg SL. TopHat2: accurate alignment of transcriptomes in the presence of insertions, deletions and gene fusions. Genome Biol. 2013;14(4):R36.

10. Anders S, Pyl PT, Huber W. HTSeq--a Python framework to work with high-throughput sequencing data. Bioinformatics. 2015;31(2):166-9.

11. Love MI, Huber W, Anders S. Moderated estimation of fold change and dispersion for RNA-seq data with DESeq2. Genome Biol. 2014;15(12):550.

12. Ross Ihaka RG. R: A Language for Data Analysis and Graphics. 1996;Journal of Computational and Graphical Statistics (3):299-314.

13. Su W, Sun J, Shimizu K, Kadota K. TCC-GUI: a Shiny-based application for differential expression analysis of RNA-Seq count data. BMC Res Notes. 2019;12(1):133.

14. Tanaka A, Woltjen K, Miyake K, Hotta A, Ikeya M, Yamamoto T, et al. Efficient and reproducible myogenic differentiation from human iPS cells: prospects for modeling Miyoshi Myopathy in vitro. PLoS One. 2013;8(4):e61540.
