# Supplementary material for: Collagen-VI supplementation by cell transplantation improves muscle regeneration in Ullrich congenital muscular dystrophy model mice
Source: Stem Cell Res Ther. 2021 Aug 9;12:446. doi: 10.1186/s13287-021-02514-3 (PMC8351132; doi:10.1186/s13287-021-02514-3)
Supplement: Supplementary file 3 — Additional file 3: Supplemental Figures and Legends. Figures S1-S10 [file 13287_2021_2514_MOESM3_ESM.pdf]

Supplemental Figures and Legends

Figure S1

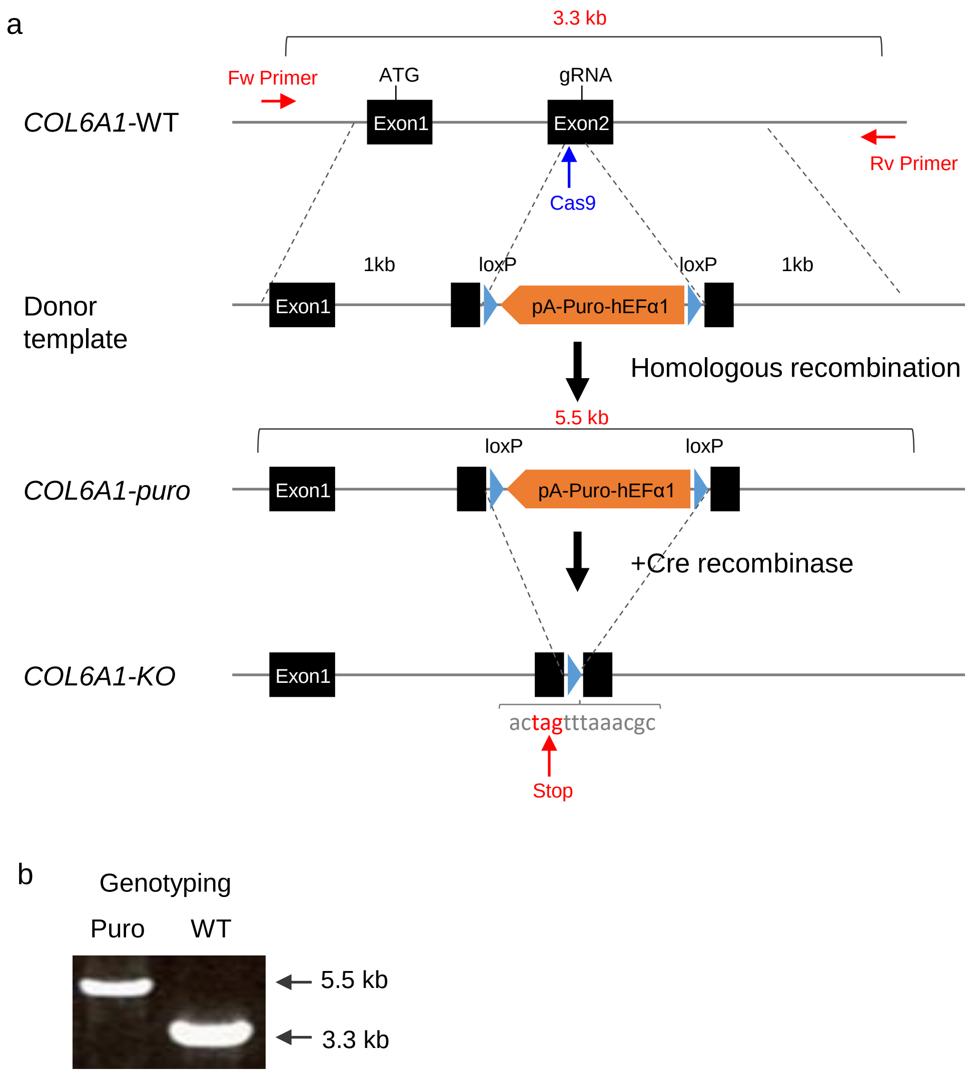

Figure S1. Generation of the *COL6A1 KO* iPSC line
a Schematic overview depicting the *COL6A1* KO cell line establishment strategy. Top line shows the *COL6A1* gene structure. The blue arrow indicates the Cas9 cut site, and the red arrows indicate the forward and reverse primers for genotyping. The homologous arms of the donor vector are indicated as the left arm (1 kb) and right arm (1 kb). Knockout cells were selected by puromycin, and the monoclonal cell population was isolated. The loxP-Puro-loxP cassette was removed by Cre recombinase.
b PCR genotyping using the primers in (A) indicates homozygous cell lines. The WT band (3.3 kb) and *COL6A1-puro* band (5.5 kb) are shown.

Figure S2

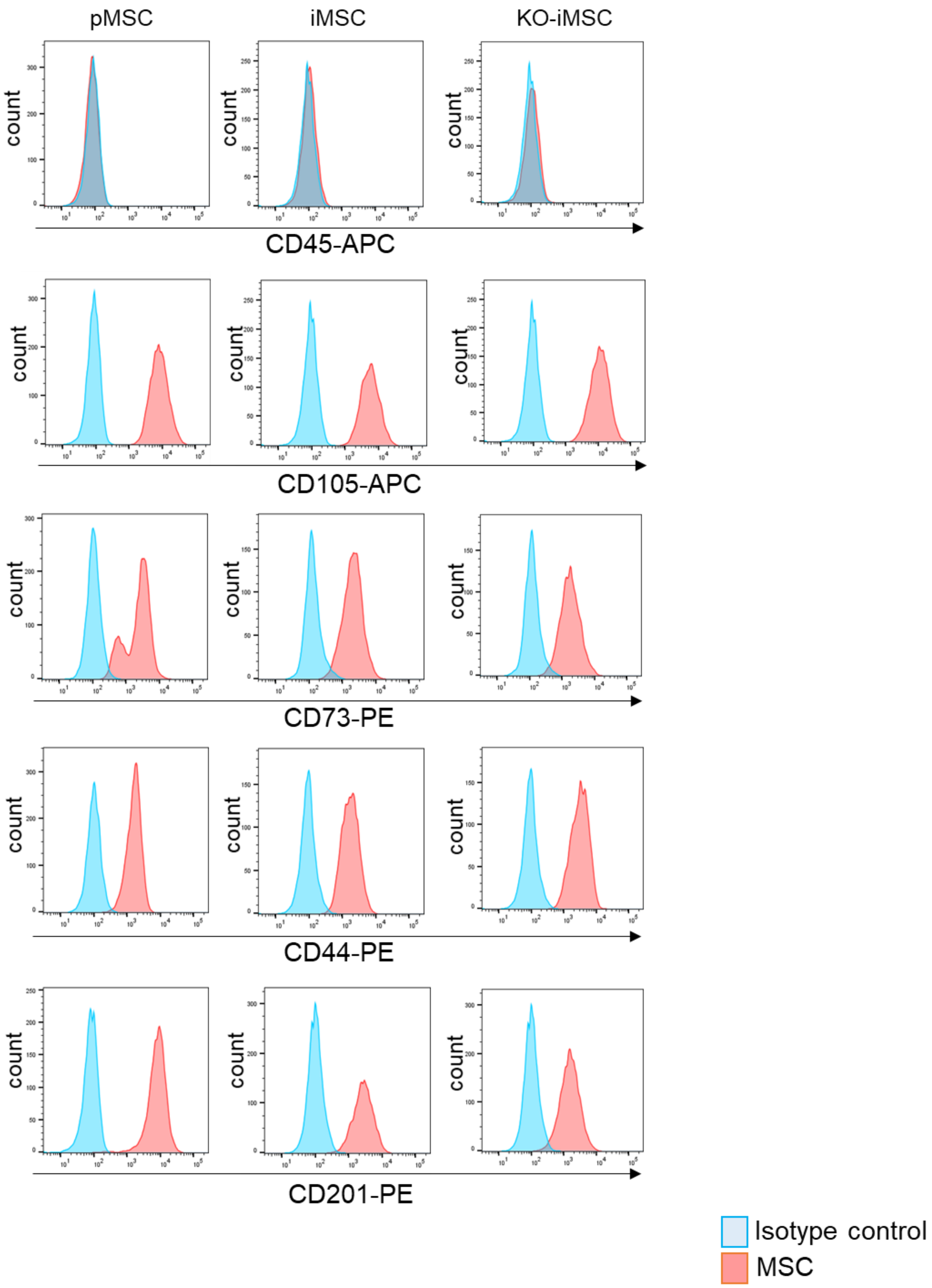

Figure S2. Flow cytometry analysis of pMSCs, iMSCs, and KO-iMSCs
Expression of MSC-related surface markers in pMSCs derived from human skeletal muscle, iMSCs, KO-iMSCs (pink), and isotype control (blue).

Figure S3

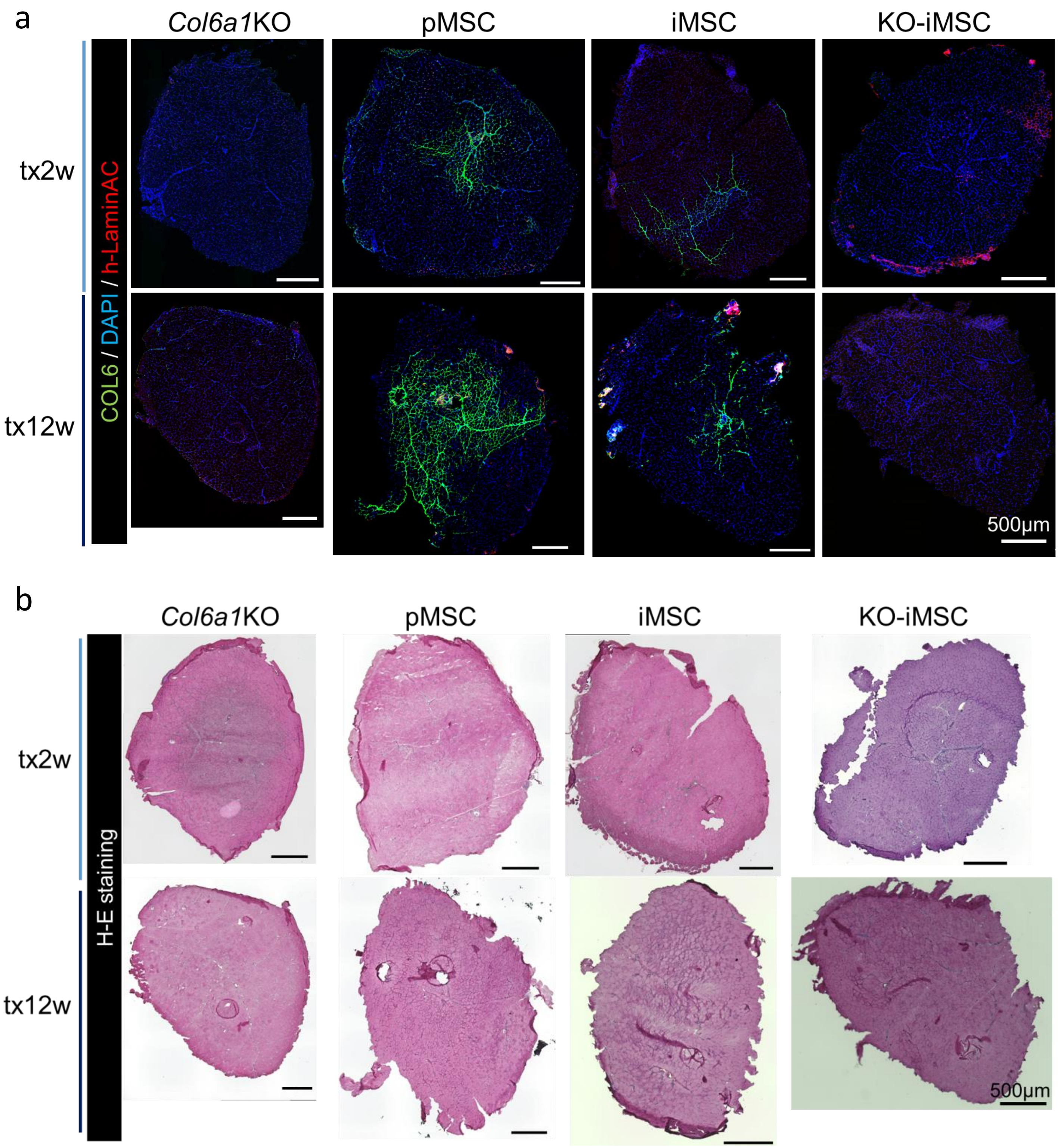

Figure S3. COL6 supplementation in whole cross-sectional areas of TA muscles transplanted with pMSCs, iMSCs, or KO-iMSCs

Sectional images of whole TA muscles 2 weeks (upper rows) and 12 weeks (lower rows) after medium injection (left) or pMSC (left middle), iMSC (right middle), or KO- iMSC (right) transplantation.

a Sections were stained with anti-human Lamin A/C antibody (red) and anti-panCOL6 antibody (green). Nuclei were stained with DAPI (blue). Scale bars, 500 μm.

b Sections were stained with H&E. Scale bars, 500 μm.

Figure S4

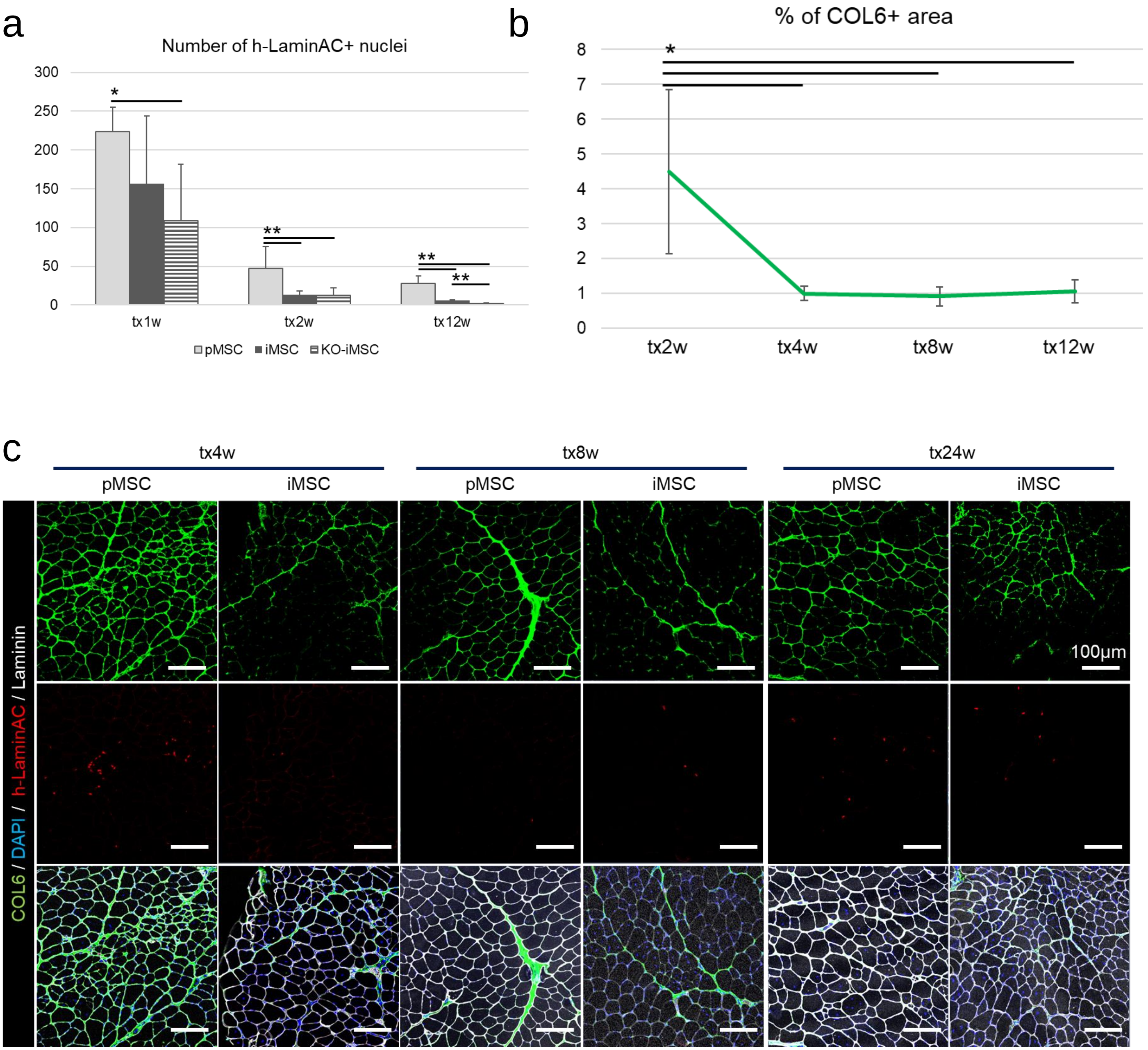

Figure S4. COL6 supplemented by pMSC or iMSC transplantation remains in TA muscle even after 4, 8, and 24 weeks

a Number of human LaminA/C+ nuclei per whole TA 1 week (tx1w), 2 weeks (tx2w) or 12 weeks (tx12w) after the transplantation. Data are the mean ± SD. For tx1w, n=6 (pMSCs, iMSCs, and KO-iMSCs each); For tx2w, n=9 (pMSCs), n=12 (iMSCs), and n=12 (KO- iMSCs); for tx12w, n=6 (pMSCs, iMSCs, and KO-iMSC). *P <0.05. **P < 0.01.

b Quantitative data of the COL6 positive area per whole iMSC-transplanted TA CSA 2 weeks (tx2w), 4weeks (tx4w), 8weeks (tx8w), or 12 weeks (tx12w) after the transplantation. Data are the mean ± SD. n=12 (tx2w), n=6 (tx4w), n=6 (tx8w), and n=6 (tx12w). *P <0.05.

c Sectional images of TA muscles 4 (left), 8 (middle), 24 (right) weeks after pMSC (left column) or iMSC (right column) transplantation. Sections were stained with anti- Laminin α2 antibody (white), anti-human Lamin A/C antibody (red), and anti-panCOL6 antibody (green). Nuclei were stained with DAPI (blue). Scale bars, 100 μm.

Figure S5

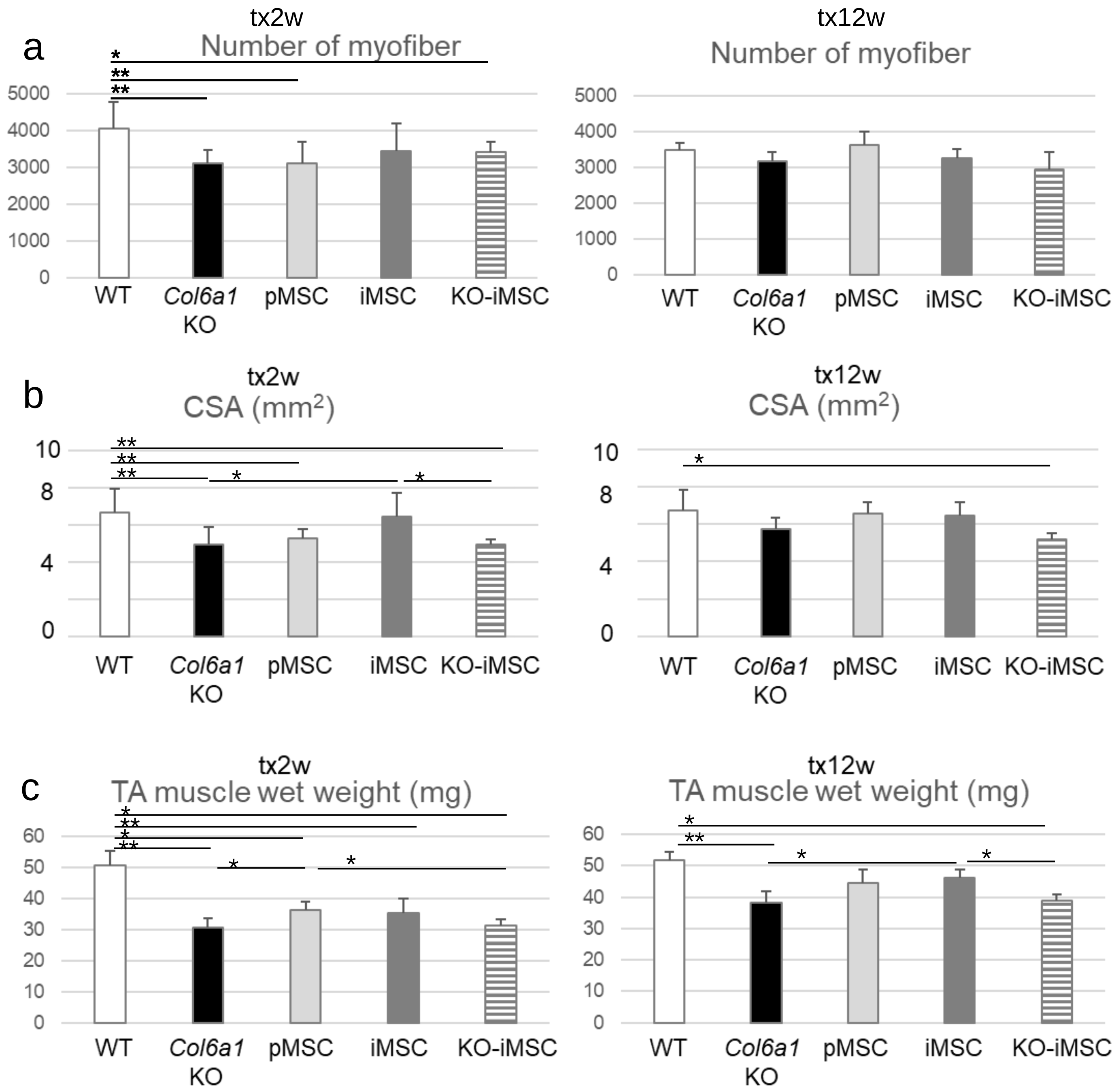

Figure S5. Cross-sectional areas and muscle wet weight of whole TA muscle transplanted with pMSCs, iMSCs, or KO-iMSCs

a Number of myofibers in TA muscles.

Data are the mean ± SD. For tx2w, n = 15 (WT), n = 10 (*Col6a1*KO), n = 9 (pMSCs), n = 12 (iMSCs), and n = 7 (KO-iMSCs); for tw12w, n = 17 (WT), n = 11 (*Col6a1*KO), n = 6 (pMSCs, iMSCs, and KO-iMSCs each). *P < 0.05. **P < 0.01.

b The CSA of whole TA muscle in *Col6a1*KO mice 2 weeks or 12 weeks after medium injection (KO) or pMSC, iMSC, or KO-iMSC transplantation and in WT mice (WT) of the same age. Data are the mean ± SD. n = 6. *P < 0.05. **P < 0.01.

c The muscle wet weight of TA muscles of *Col6a1*KO mice 2 weeks or 12 weeks after medium injection or pMSC, iMSC, or KO-iMSCs transplantation and of WT mice of the same age. Data are the mean ± SD. n = 6. *P < 0.05. **P < 0.01.

Figure S6

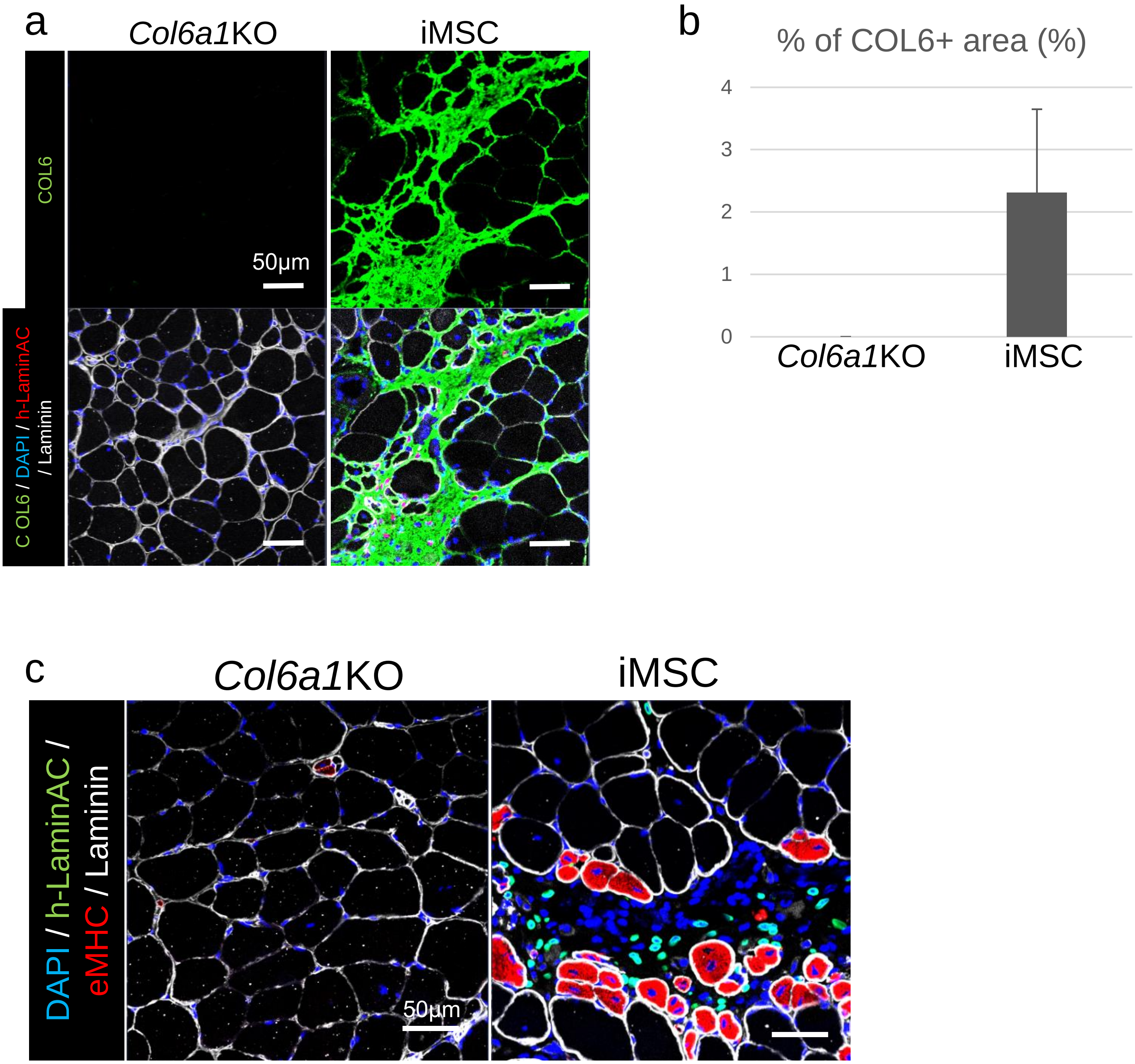

Figure S6. COL6 supplementation in adult *Col6a1*KO mice

a Sectional images of TA muscles 2 weeks after medium injection (left column) or iMSC transplantation (right column). Sections were stained with anti-Laminin α2 antibody (white), anti-human Lamin A/C antibody (red), and anti-panCOL6 antibody (green). Nuclei were stained with DAPI (blue). Scale bars, 50 μm.

b Quantitative data of the COL6-positive area per whole TA CSA 2 weeks after the transplantation. Data are the mean ± SD. n = 3.

c Sectional images of TA muscles 1 week after medium injection (left column) or iMSC transplantation (right column). Sections were stained with anti-eMHC antibody (red), anti-Laminin α2, and anti-human Lamin A/C antibody (green). Nuclei were stained with DAPI (blue). Scale bars, 50 μm.

Figure S7

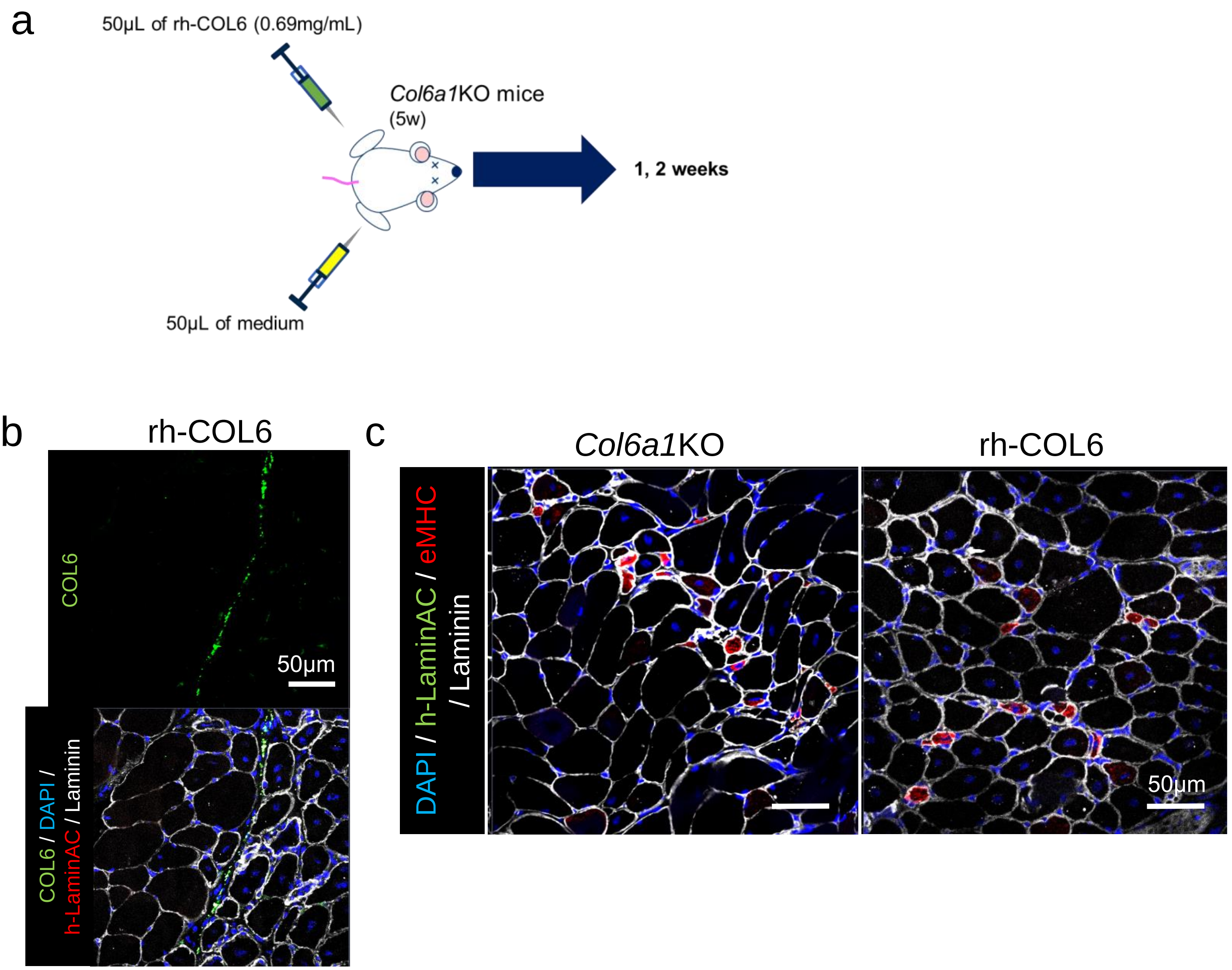

Figure S7. rh-COL6 injection into the TA muscles of *Col6a1*KO mice
a Schematic representation of injecting rh-COL6 into the TA muscles of *Col6a1*KO mice.
b Sectional images of the TA muscles 1 week after rh-COL6 (upper) or medium (lower) injection. Sections were stained with anti-Laminin α2 antibody (white), anti-human Lamin A/C antibody (red), and anti-panCOL6 antibody (green). Nuclei were stained with DAPI (blue). Scale bars, 50 μm.
c Sectional images of the TA muscles 1 week after medium (left) or rh-COL6 (right) injection. Sections were stained with anti-eMHC antibody (red), anti-Laminin α2, and anti-human Lamin A/C antibody (green). Nuclei were stained with DAPI (blue). Scale bars, 50 μm.

Figure S8

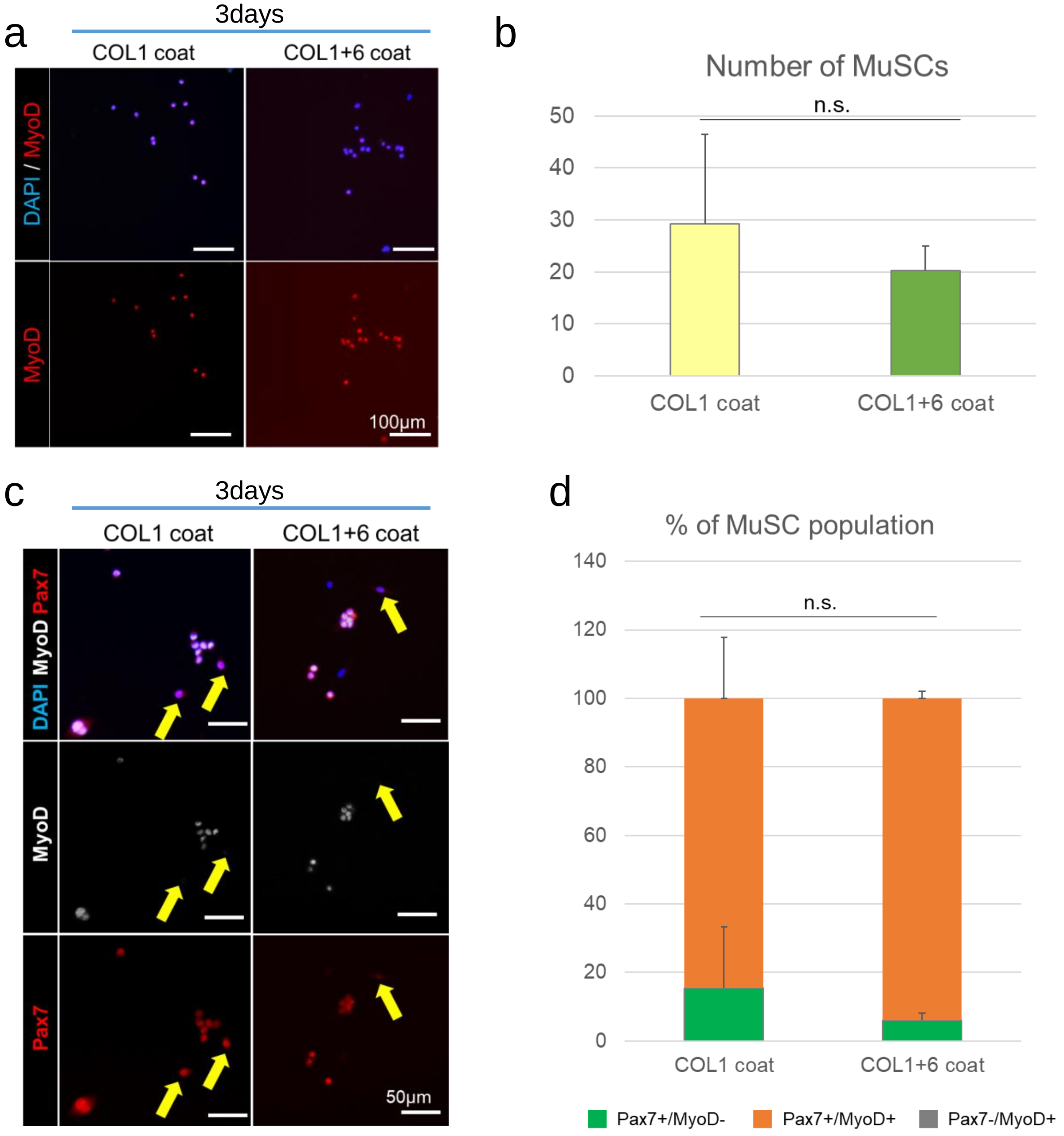

a
3days
COL1 coat
COL1+6 coat
DAPI / MyoD
MyoD
100μm
b
Number of MuSCs
n.s.
50
40
30
20
10
0
COL1 coat
COL1+6 coat
c
3days
COL1 coat
COL1+6 coat
DAPI MyoD Pax7
MyoD
Pax7
50μm
d
% of MuSC population
140
n.s.
120
100
80
60
40
20
0
COL1 coat
COL1+6 coat
Pax7+/MyoD-
Pax7+/MyoD+
Pax7-/MyoD+

Figure S8

e

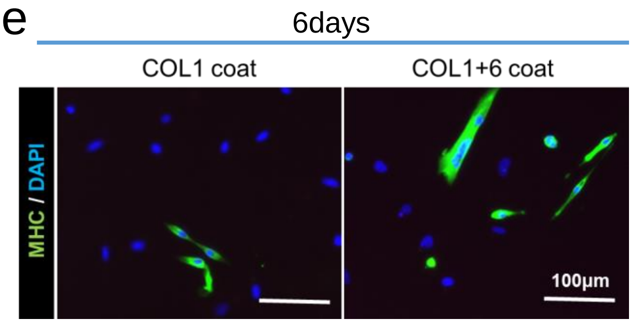

f

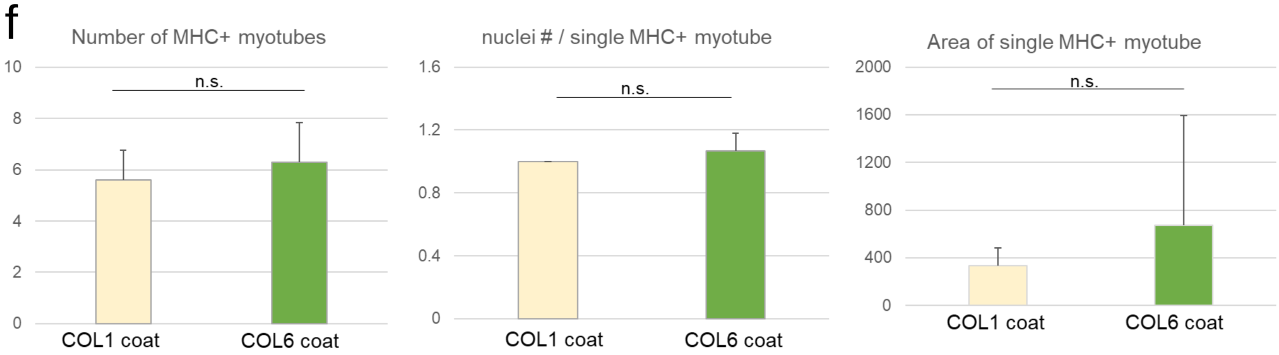

Figure S8. COL6-coating did not affect MuSC proliferation or maturation *in vitro*

a Immunofluorescence images of *Col6a1*KO mouse-derived MuSCs 3 days after culture with COL1-coating (left column) or COL1+COL6-coating (right column). Cells were stained with anti-MyoD antibody (red). Nuclei were stained with DAPI (blue). Scale bars, 100 μm.

b Number of DAPI+ mouse MuSCs 3 days after culture. Data of three independent experiments are shown as the mean ± SD. n.s.: not significant.

c Immunofluorescence images of *Col6a1*KO mouse-derived MuSCs 3 days after culture with COL1-coating (left column) or COL1+COL6-coating (right column). Cells were stained with anti-MyoD antibody (white) and anti-Pax7 antibody (red). Nuclei were stained with DAPI (blue). Scale bars, 50 μm.

d Quantification of Pax7+MyoD–, Pax7+MyoD+ and Pax7–MyoD+ cell populations 3 days after the culture. Data of three independent experiments are shown as the mean ± SD. n.s.: not significant.

e Immunofluorescence images of *Col6a1*KO mouse-derived MuSCs 6 days after culture with COL1-coating (left column) or COL1+COL6-coating (right column). Cells were stained with anti-MHC antibody (green). Nuclei were stained with DAPI (blue). Scale bars, 100 μm.

f Number of MHC+ myotubes derived from mouse MuSCs (left), number of nuclei in MHC+ myotubes derived from mouse MuSCs (middle), and area of MHC+ myotubes derived from mouse MuSCs 6 days after co-culture. Data of three independent experiments are shown as the mean ± SD. n.s.: not significant.

Figure S9

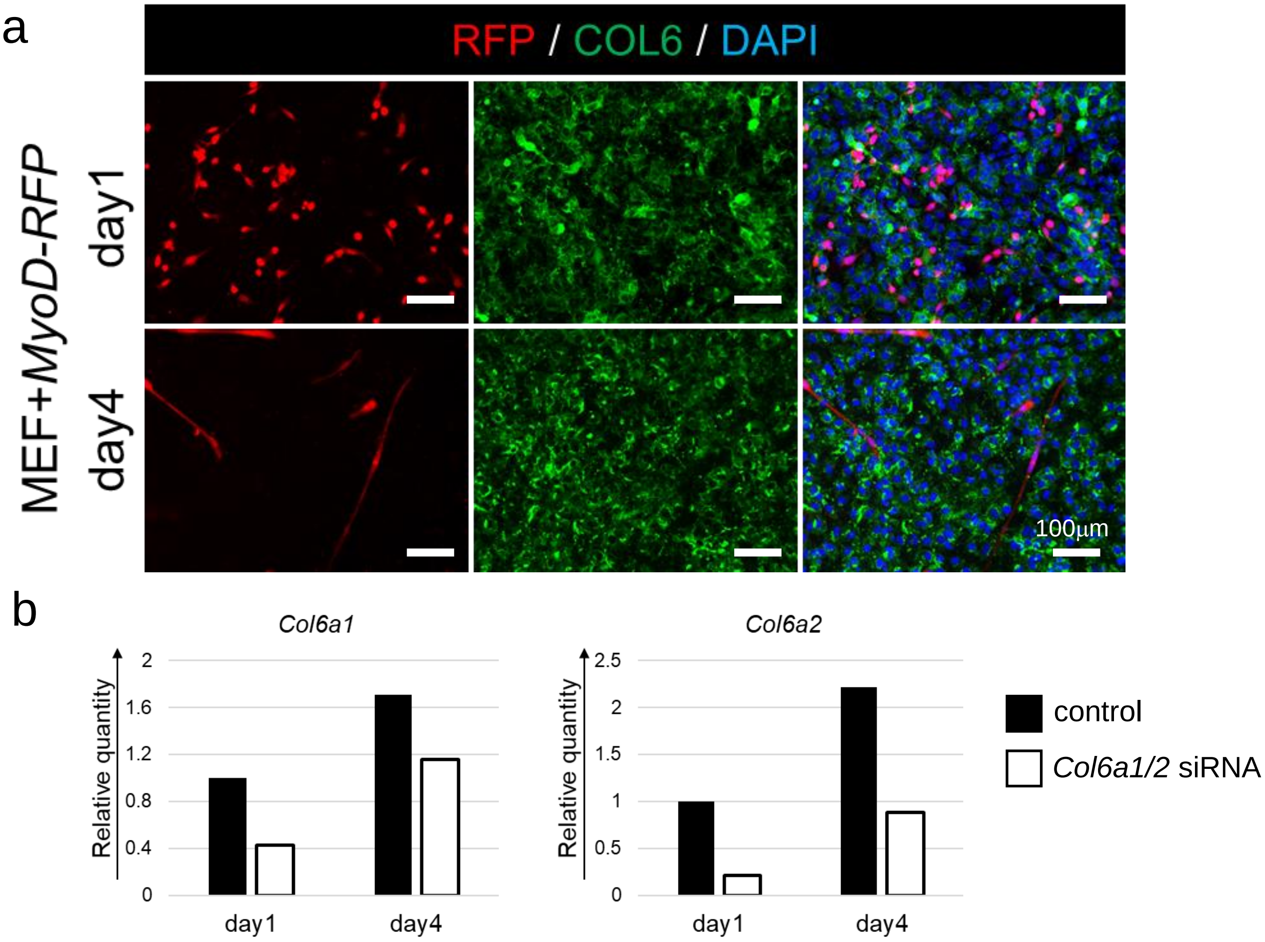

a
RFP / COL6 / DAPI
MEF+MyoD-RFP
day1
day4
100µm
b
Col6a1
Col6a2
Relative quantity
2
1.6
1.2
0.8
0.4
0
2.5
2
1.5
1
0.5
0
day1
day4
control
Col6a1/2 siRNA

Figure S9

c

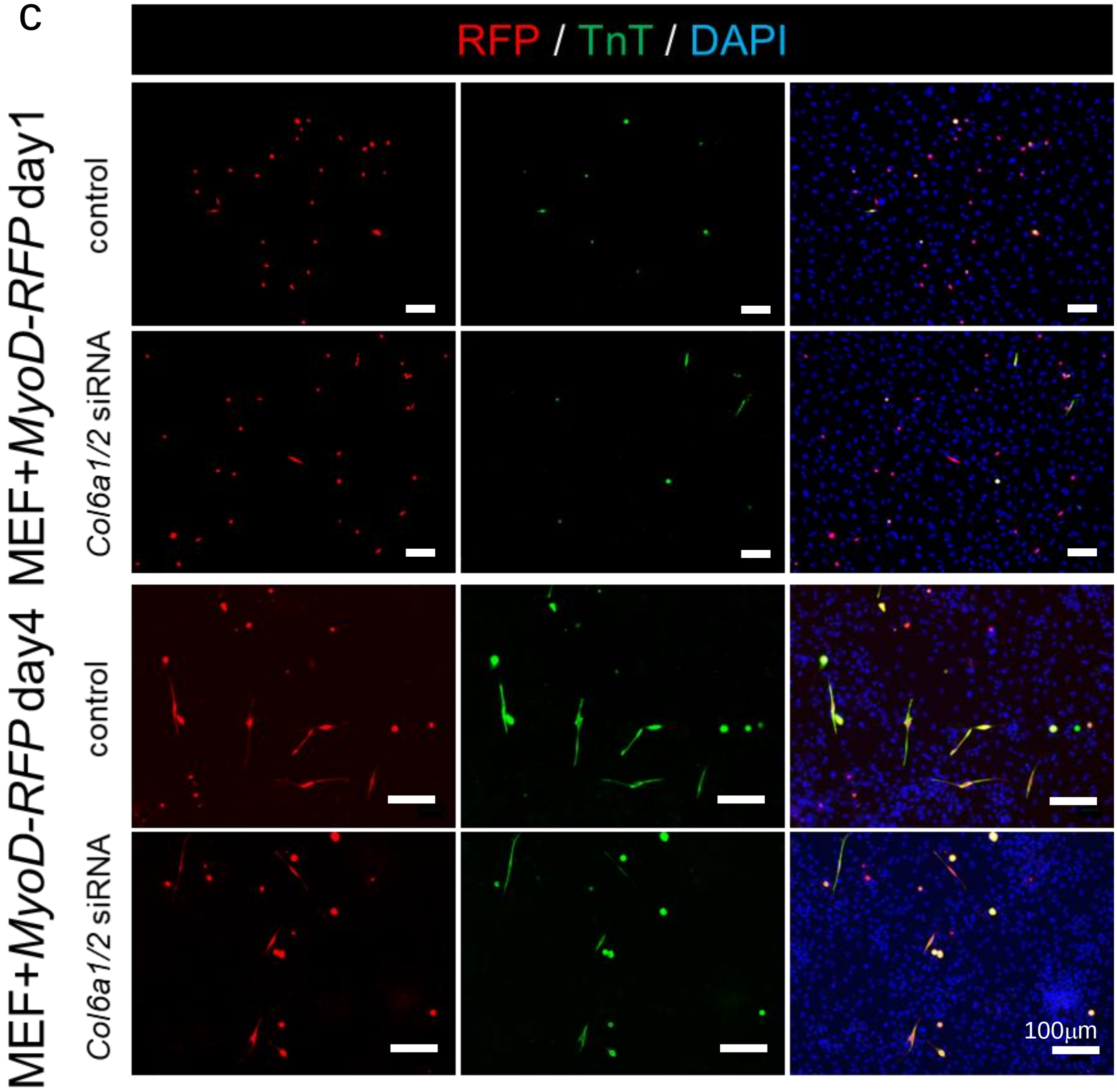

Figure S9. Co-culture of *Col6a1,2*KD-MEFs and myoblast cells

a Immunofluorescence images of *MyoD-RFP* mouse-derived MyoD+ myoblasts (red) 1 day (upper low) and 4 days (lower low) after co-culture with MEFs derived from the same mice. Cells were stained with anti-RFP antibody (red) and anti-COL6 antibody (green). Nuclei were stained with DAPI (blue). Scale bars, 100 µm.

b Gene expressions. The mRNA expression of each gene was analyzed by RT-qPCR in MyoD+ myoblasts derived from *MyoD-RFP* mice 1 day and 4 days after treatment with *Col6a1/2* siRNA. Expressions are relative to values in MyoD+ myoblasts without siRNA treatment. Data are the mean. n = 2.

c Immunofluorescence images of MyoD-RFP mouse-derived MyoD+ myoblasts (red) 1 day (upper 2 rows) and 4 days (lower 2 rows) after co-culture with MEFs derived from the same mice. *Col6a1/2* siRNA treatment: second and fourth rows; no *Col6a1/2* siRNA treatment: first and third rows. Cells were stained with anti-TnT antibody (green) and anti-RFP antibody (red). Nuclei were stained with DAPI (blue). Scale bars, 100 µm.

Figure S10

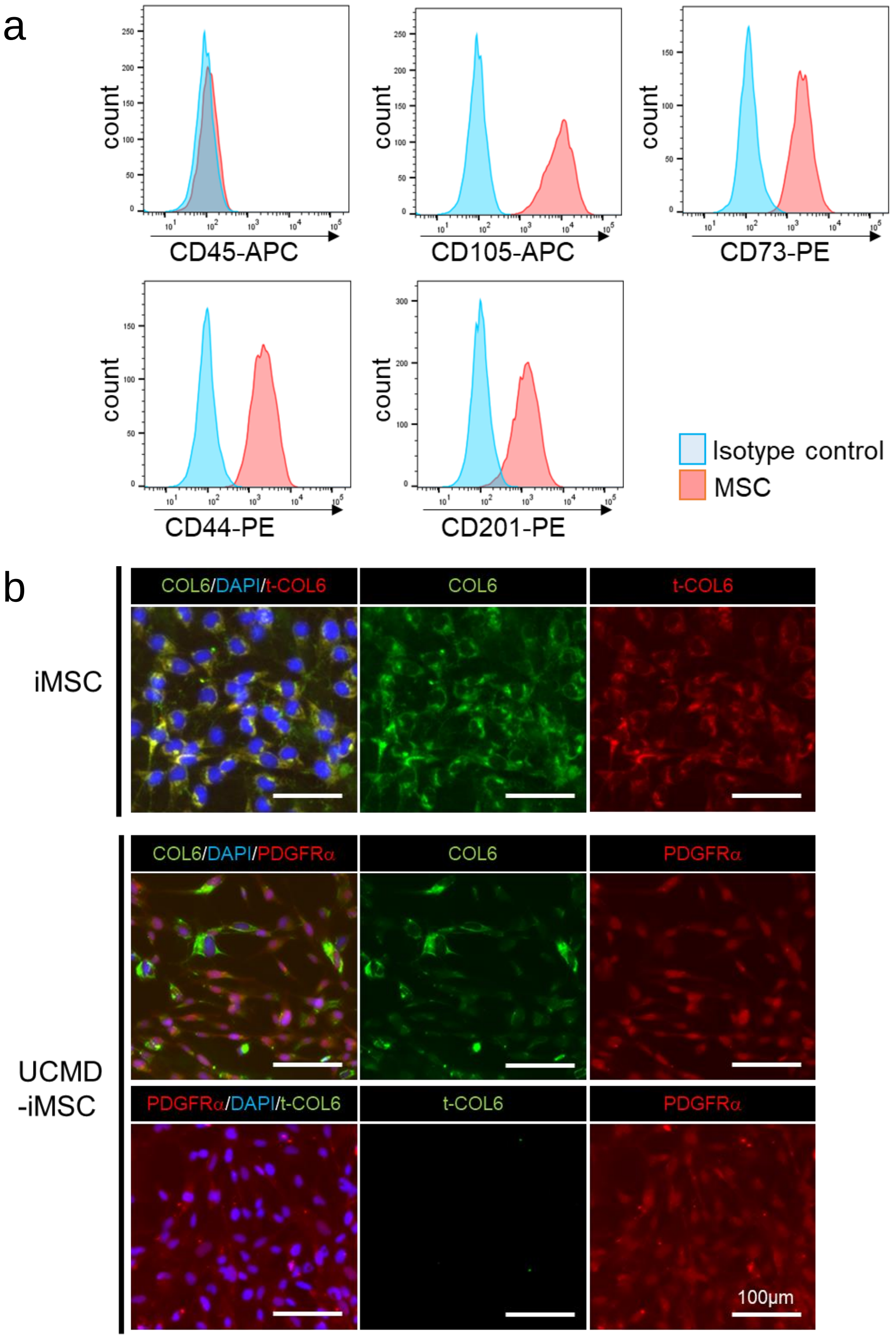

Figure S10. Characterization of UCMD patient-derived iMSCs
a Expression of MSC-related surface markers in UCMD-iMSCs (pink) and isotype control (blue).
b Immunofluorescence images of iMSCs and UCMD-iMSCs. (upper row) iMSCs were stained with anti-panCOL6 antibody (green) and anti-t-COL6 antibody (red); (middle row) UCMD-iMSCs were stained with anti-panCOL6 antibody (green) and anti-PDGFRα antibody (red); and (lower row) UCMD-iMSCs were stained with anti-t-COL6 antibody (green) and anti-PDGFRα antibody (red). Nuclei were stained with DAPI (blue). Scale bars, 100 μm.
